# Engineering of Multi‐Dynamic Bonds Toward Room‐Temperature Self‐Healing Epoxy/MXene Adaptable Network with Record‐High Toughness

**DOI:** 10.1002/advs.202508780

**Published:** 2025-06-23

**Authors:** Xiaobo Zhu, Yu Hao, Liang‐Feng Huang, Haichao Zhao, Liping Wang

**Affiliations:** ^1^ State Key Laboratory of Advanced Marine Materials Ningbo Institute of Materials Technology and Engineering Chinese Academy of Sciences Ningbo 315201 China; ^2^ University of Chinese Academy of Sciences Beijing 100049 China; ^3^ Research Center for Advanced Interdisciplinary Sciences Ningbo Institute of Materials Technology and Engineering Chinese Academy of Sciences Ningbo 315201 China

**Keywords:** bioinspired, epoxy/MXene adaptable network, self‐healing, Ti_3_C_2_T_x_ MXene, toughness

## Abstract

Epoxy thermosets with exceptional toughness and self‐healing properties are essential for high‐end manufacturing applications and sustainable development. Conventional epoxy‐crosslinked networks are inherently brittle, leading to a lack of these traits. Drawing inspiration from mussel nacre and byssus, a bionic epoxy/MXene network is developed, featuring a multi‐type dynamic bond system and an inverse‐artificial nacre structure. Through hierarchical assembly, the network integrates side‐chain quadruple hydrogen bonds (H‐bonds), dynamic disulfide bonds, and interfacial H‐bonds, facilitating rapid energy dissipation. This epoxy demonstrates exceptional performance through meticulous engineering, exhibiting impressive toughness (210.75 MJ m^−3^), exceeding that of spider silk by 30%. It also shows remarkable stretchability (864.72%) and rapid self‐healing capabilities (90.0% recovery within 2 h at 25 °C). This combination of these properties is consistently maintained under various environmental conditions due to the protection of the dynamic bonds by the hydrophobic chains. Furthermore, the bionic network enhanced the composites with superior gas impermeability and high interfacial adhesive strength (9.58 MPa). This study offers novel insights into the development of high‐performance, durable protective coatings, and flexible devices designed for enhanced tolerance to harsh marine environments.

## Graphical abstract

Inspired by mussel nacre and byssus, a multi‐type dynamic chemically bonded epoxy/MXene adaptable network is fabricated through a hierarchical assembly strategy, establishing the toughest (210.75 MJ m^−3^) room‐temperature self‐healing (90.04% recovery within 2 h at 25 °C) epoxy resin to date, challenging the limitations of conventional thermosetting resin with low toughness and difficulty in self‐healing local damage defects.

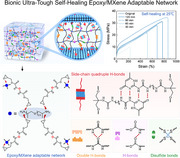

## 1 Introduction

Epoxy (EP) thermosetting resins are extensively employed in coatings, adhesives, and structural components because of their exceptional mechanical strength, corrosion resistance, and superior thermal properties.^[^ ^1^, ^2^, ^3^, ^4^, ^5^, ^6^, ^7^ ^]^ Developing ultra‐tough epoxy resins capable of autonomously repairing damage and defects at room temperature has become a key focus in high‐end intelligent manufacturing. These resins can extend the material's lifespans, enhance the reliability and durability of functional devices, and contribute to environmental sustainability.^[^ ^8^, ^9^, ^10^, ^11^ ^]^ However, the inherent conflict between strength, toughness, and self‐healing ability, resulting from the covalent network structure of epoxy thermosetting resins, has significantly limited their wider application in high‐performance composites. Typically, their extension at break ranges from 1% to 10%, with tensile toughness less than 5.0 MJ m^−3^.^[^ ^2^ ^]^ For decades, researchers have been trying to enhance the toughness of epoxy resins.^[^ ^12^, ^13^, ^14^, ^15^ ^]^ However, developing strategies that simultaneously enhance the mechanical strength and toughness of thermosetting resins remains a significant challenge.

The simultaneous presence of high strength and toughness is a characteristic frequently observed in biological systems. Biological tissues, such as mussel nacre and byssus, which rely on non‐covalent assembly, demonstrate an extraordinary combination of contrasting mechanical strength, toughness, and self‐healing ability following damage, effectively meeting the previously mentioned material requirements.^[^ ^16^, ^17^, ^18^, ^19^ ^]^ The “brick–and–mortar” structure of mussel nacre consists primarily of inorganic flakes, with ≈4 wt.% biopolymers, exhibiting exceptional strength, toughness, and stability.^[^ ^20^, ^21^ ^]^ Unlike conventional adhesives, mussel byssus achieves high toughness, self‐healing ability, and adhesive strength through a unique mechanism, using non‐covalent interactions, particularly hydrogen bonding between adhesive proteins on the byssal threads and the biological substrate.^[^ ^22^, ^23^ ^]^ Therefore, incorporating reversible dynamic bonds into polymer networks and adding rigid inorganic nanofillers are key approaches for enhancing the strength and toughness of materials.

Motivated by this concept, a considerable number of studies have been conducted to achieve enhanced performance through the introduction of nanofillers or the construction of covalent adaptable networks.^[^ ^24^, ^25^, ^26^, ^27^ ^]^ Covalent adaptive networks are a type of polymers crosslinked by reversible covalent bonds, including disulfide bonds, oxime‐carbamate bonds, and Diels‐Alder adducts, etc.^[^ ^28^, ^29^, ^30^ ^]^ It is capable of dynamic bond dissociation or reversible crosslinking under specific conditions, offering self‐healing and plasticity similar to those of thermoplastic resins. However, in conventional covalent adaptive networks, self‐healing under mild conditions is difficult because of the strong dynamic covalent bonds with high bond energies. In addition, the incorporation of nanofillers restricts the mobility of the polymer molecular chains, leading to limited self‐repair properties and challenges in simultaneously optimizing the toughness and self‐healing capability.

In recent years, ultra‐thin 2D titanium carbide (Ti_3_C_2_T_x_) MXene nanosheets have garnered significant attention for their outstanding mechanical properties, exceptional electrical conductivity, and tunable surface chemistry.^[^ ^31^, ^32^, ^33^ ^]^ However, the high stiffness of Ti_3_C_2_T_x_ and its limited interfacial compatibility with polymer matrices hinder its effective use in practical applications. Currently, effective methods for integrating these rigid 2D materials with polymer networks to create self‐healing materials with high mechanical properties remain challenging. Biological tissues in nature serve as invaluable models for the structural design of advanced materials. Combining non‐covalent bonds with thoughtfully designed biomimetic structures is a widely adopted approach for creating self‐healing materials and flexible devices.

This study introduces a hierarchical assembly strategy, inspired by mussel nacre and byssus, to develop ultra‐tough self‐healing epoxy resins. The approach involves the non‐covalent bond‐driven integration of MXene nanosheets into multi‐type dynamic adaptable networks to build inverse‐artificial (IA)‐nacre structures. Initially, flexible side‐chain quadruple H‐bonds and dynamic disulfide (S─S) bonds are incorporated into the molecular chains to improve chain mobility and modulate mechanical properties. Subsequently, 2‐ureido‐4[1H]‐pyrimidinone (UPy)‐modified MXene nanosheets, featuring quadruple H‐bonds, were incorporated to introduce high‐density interfacial H‐bonds. These facilitate rapid energy dissipation, yielding composites with markedly enhanced toughness and accelerated self‐healing capabilities under low temperature, room‐temperature, and aqueous solutions. The material exhibits a maximum elongation at break (864.72%) and a toughness (210.75 MJ m^−3^), which are improved by two orders of magnitude greater than those of traditional epoxy resins. Furthermore, the biomimetic network imparts exceptional gas impermeability and robust interfacial adhesive strength to the composites, while the integrated flexible sensor devices demonstrate outstanding stability. The proposed findings indicate that these high‐performance self‐healing materials hold significant potential for applications in metal corrosion protection and flexible functional devices under intricate marine environments.

## 2 Results and Discussion

### 2.1 Design and Preparation for Epoxy/MXene Adaptable Network

**Scheme 1:**
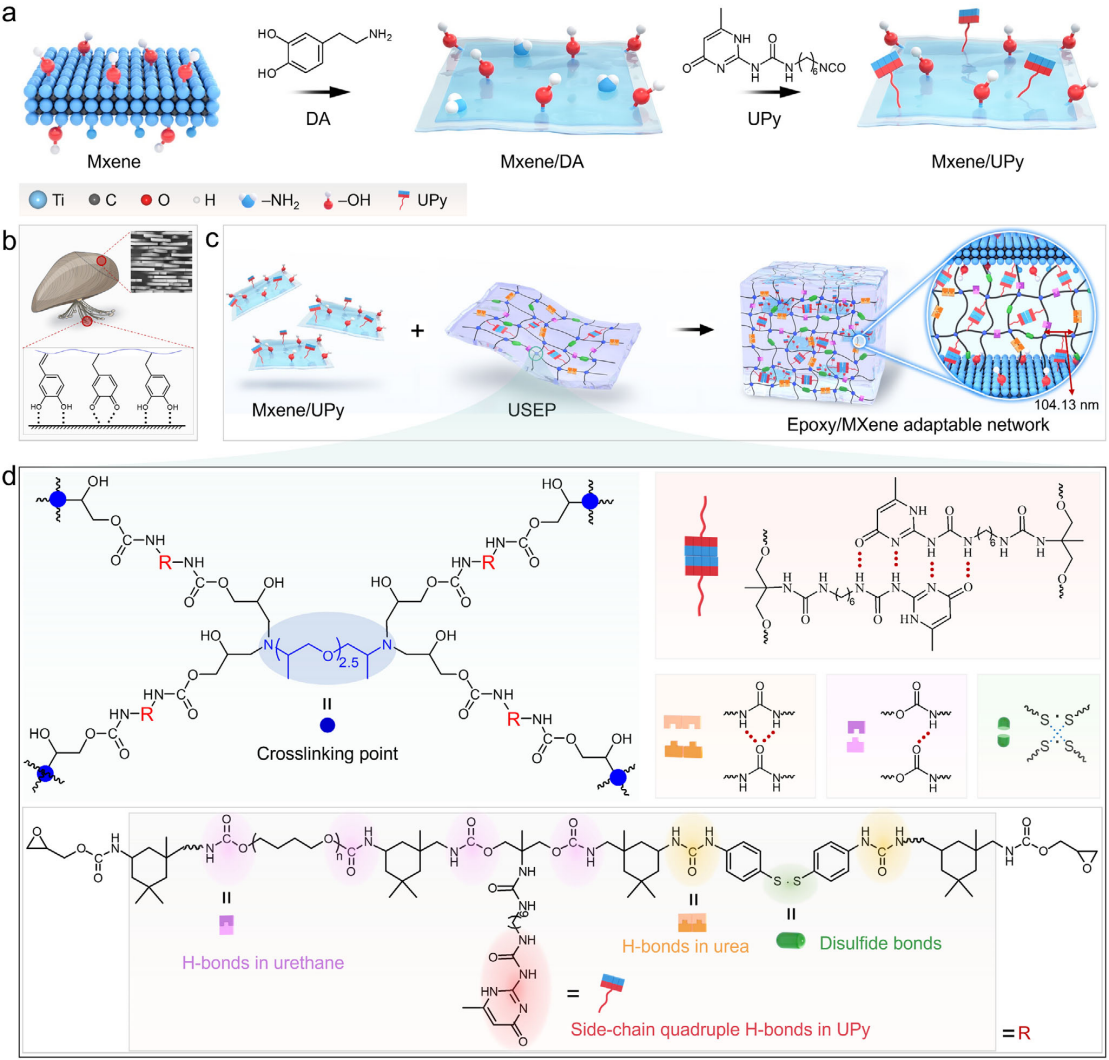
a) Preparation process of the MXene/UPy nanosheets. b) Mussel nacre and byssus structures. c) Schematic representation of the epoxy/MXene adaptable network featuring multi‐type dynamic bonds and inverse‐artificial nacre structure. d) Interaction of dynamic bonds within the composite.

Non‐covalent bond‐driven self‐assembled UPy‐modified MXene (MXene/UPy) 2D nanosheets (**Scheme** **1**a) were embedded into epoxy matrices containing multi‐type dynamic chemical bonds, mimicking the nacre and byssus structure of mussels (Scheme 1b) to design strong and tough self‐healing epoxy/MXene adaptable networks (Scheme 1c,d). For the multi‐type dynamic epoxy molecular chain network, the preparation process and route are shown in the **Supporting Information methods section** and Figures S1–S3 (Supporting Information). Specifically, through a hierarchical assembly strategy, side‐chain quadruple H‐bonds and S─S bonds are first introduced into the molecular network to increase the density of dynamic chemical bond receptors and donors, and promote the rapid reconstruction of broken chemical bonds. Then, an epoxy covalent network was constructed (referred to as USEP), which combined with hydrophobic chain segments to protect the movement‐reconstruction activities of H‐bonds and S─S bonds at low temperature and in aqueous environments.

Based on the dynamic chemical bond enhancement technique applied to the surface of the MXene/UPy 2D nanosheets (Scheme 1a), dopamine (DA) was first coated onto the surface of MXene (MXene/DA). This introduced an amine and multi‐hydroxyl structure, resembling that of a mussel byssus, to enhance the interfacial compatibility between the nanosheets and the epoxy matrix. MXene/UPy 2D nanosheets containing a quadruple H‐bond were synthesized through the reaction of the amino group on the surface of MXene/DA with UPy. This process was employed to regulate the density of the interfacial H‐bond acceptors and donors.

The introduction of a small amount of MXene/UPy nanosheets into the epoxy crosslinked network facilitated the production of abundant interface quadruple H‐bonds. This was attributed to the non‐covalent interactions between the UPy moieties on the nanosheets and the side chains of the epoxy molecules. The parallel alignment of the 2D nanosheets within the polymer was achieved using an evaporation‐induced assembly method with controlled interfacial H‐bonds cross‐linking and curing rates, resulting in the formation of an IA‐nacre structure with a composition that contrasts with that of the natural nacre. The epoxy/MXene adaptable network, carefully designed to include side‐chain quadruple H‐bonds, abundant interfacial H‐bonds, and dynamic S─S bonds, is labeled as USEP‐M*_x_*, where *x* represents the mass percentage of the MXene/UPy nanosheets to the USEP resin. The combined effect of the multi‐type dynamic chemical bonds within the adaptable network and the IA‐nacre structure enhances and toughens the resin, while also imparting strong mobility to the molecular chains, enabling rapid and autonomous self‐repair of the epoxy/MXene adaptable network upon damage.

### 2.2 Characterization and Validation of Epoxy/MXene Adaptable Network

**Figure 1:**
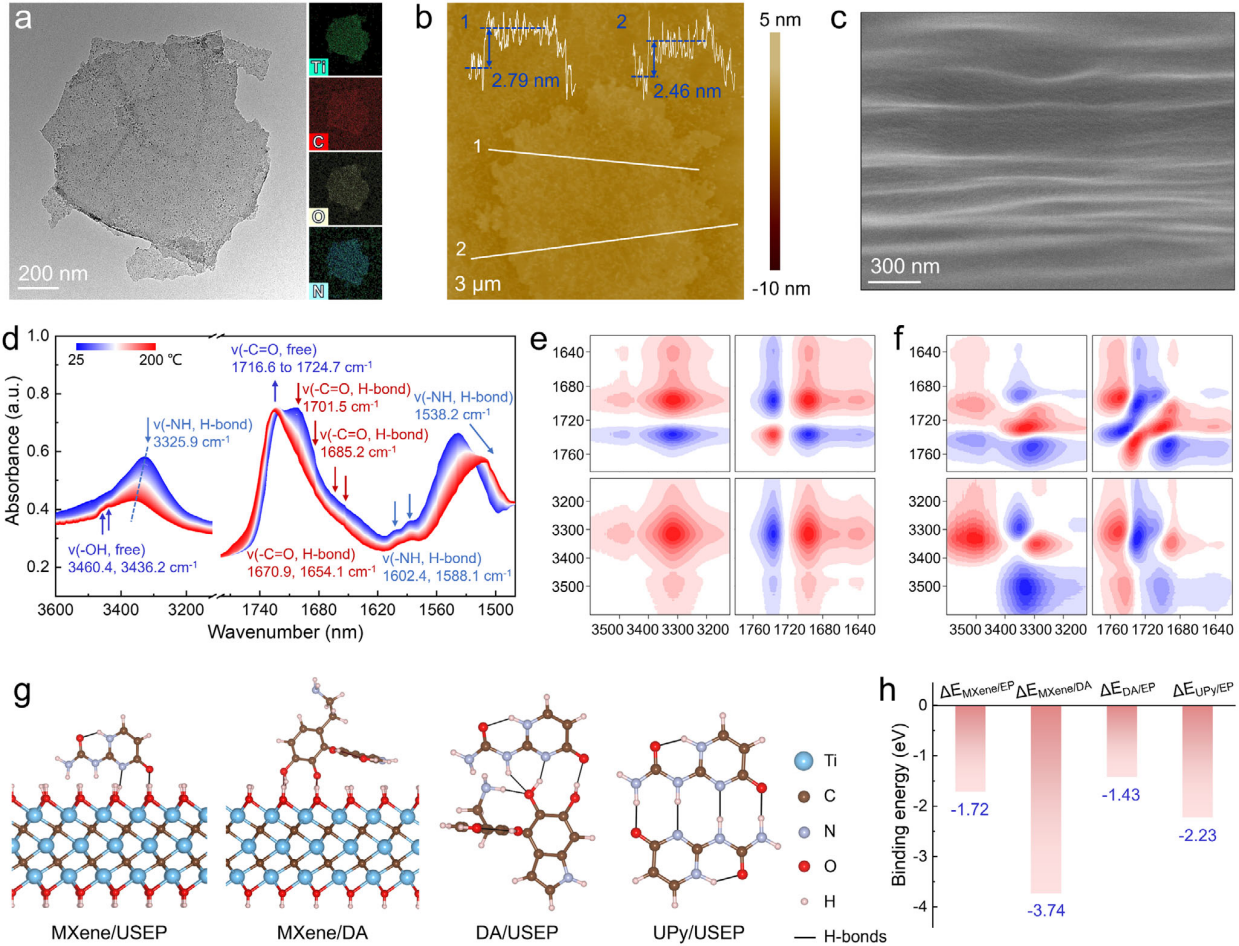
a) TEM image and the corresponding elemental distribution map of the MXene/UPy nanosheets. b) SPM image of the MXene/UPy nanosheets. c) SEM image of the USEP‐M_0.5_ adaptable network. d) Temperature‐dependent IR spectra of USEP‐M_0.5_ and the associated 2DCOS analysis: e) synchronous and f) asynchronous spectra recorded during heating from 25 to 200 °C (where red and blue in the 2DCOS plots indicate positive and negative intensities, respectively). g) Bonding modes of H‐bonds and (h) binding energy between epoxy and nanosheets before and after modification.

**Figure** **1**a and Figures S4 and S5 (Supporting Information) present the transmission electron microscopy (TEM) images of the nanosheets, both before and after modification. It exhibited a uniform distribution of fine particles on the MXene/UPy surface, accompanied by an increase in the content of carbon (C), oxygen (O), and nitrogen (N) elements. The scanning probe microscopy (SPM) analysis revealed that the thickness of the nanosheets increased to 2.79 nm (Figure 1b; Figure S6, Supporting Information). This indicates that the modified MXene/UPy nanosheets maintain the high specific surface area and aspect ratio of the original MXene, while effectively filling the surface defects. This enhancement contributes to the improved gas impermeability and barrier properties of the nanosheets.

The effective grafting of the DA and UPy was demonstrated by the Fourier transform infrared (FT‐IR) spectrum and thermogravimetric (TG) analysis of the samples (Figures S7 and S8, Supporting Information). The calculated loadings of DA and UPy were ≈11.54% and 6.67%, respectively, indicating the successful incorporation of polyhydroxyl groups and abundant H‐bonds. This facilitates the formation of interface H‐bonds within the epoxy/MXene adaptable network while enhancing its adhesion properties to the substrate. Therefore, the nanosheets exhibited outstanding dispersion stability in aqueous solutions (Figure S9, Supporting Information). These results suggest that the incorporating of MXene/UPy nanosheets will effectively improve the mechanical properties and impermeability of the epoxy/MXene adaptable networks.

Figure 1c shows the SEM cross‐sectional morphology image of the USEP‐M_0.5_ adaptable network. The MXene/UPy nanosheets align parallel to each other within the epoxy resin, creating a bionic inverse‐artificial nacre structure. This arrangement effectively can resist the propagation of the applied stress and cracks, thereby improving the mechanical strength and toughness of the epoxy/MXene adaptable network. Furthermore, the composites exhibited outstanding thermal stability (Figure S10, Supporting Information). A gradual increase in the thermal decomposition temperature of the composites was observed with increasing content of the MXene/UPy nanosheets. This phenomenon can be ascribed to the formation of interfacial H‐bonds, which significantly strengthen the intermolecular forces within the composite matrix.

Differential scanning calorimetry (DSC) analysis (Figure S11, Supporting Information) of the materials revealed the presence of two distinct glass transition temperatures (*T_g_*) for USEP at −71.29 and 20.32 °C. These two *T_g_* values strongly indicate the existence of two heterogeneous phases within the USEP material, likely resulting from the intermixing of multiple types of chemical bond segments and the epoxy crosslinked network. This observation indicates the formation of an epoxy/MXene adaptable network. Upon embedding the MXene/UPy nanosheets, the *T_g_* of the epoxy crosslinked network decreased to 12.89 °C. This shows that the incorporation of the rigid nanosheets interferes with the formation of the epoxy covalent network and decreases the crosslink density. At this point, the sacrifice in the mechanical strength attributed to the disruption of the epoxy covalent network is compensated for by a notable increase in the material's toughness and tensile properties. Ultimately, the introduction of the MXene/UPy nanosheets and the subsequent generation of the interfacial H‐bonds resulted in a synergistic enhancement of the strength and toughness properties of the epoxy/MXene adaptable network. At the same time, the mobility of the molecular chains at room temperature increased.

Infrared (IR) spectroscopy was employed to research the chemical bond interactions between the molecules of the composite, with the results shown in Figure S12 (Supporting Information). The structural compositions of the four epoxy resins were similar. The intensity of the H‐bonds in the 1550–1800 cm^−1^ range increased with the higher content of the MXene/UPy nanosheets, indicating an enhancement in the intermolecular force. To further confirm the formation of H‐bonds in the epoxy/MXene adaptable network, in situ temperature‐dependent IR spectra were used to monitor the structural changes in the USEP‐M_0.5_ elastomer (Figure 1d). The intensities of the H‐bonded –C═O stretching vibrations in the polymer network, observed at 1701.5, 1685.2, 1670.9, and 1654.1 cm^−1^ for the UPy, urethane, and urea groups, all decreased upon heating from 25 to 200 °C. The free –C═O stretching vibration moved from 1716.6 to 1724.7 cm^−1^ accompanied by an increase in intensity.

The –N–H group in the H‐bonds shows a comparable pattern of change. The bending and stretching vibrations of –N–H were observed to move from 1538.2 to 1508.6 cm^−1^ and from 3325.9 to 3354.8 cm^−1^, respectively, while the intensity of the free –OH group, located at 3460.4 and 3436.2 cm^−1^ increases. These findings indicate the development of high‐density H‐bonds within the epoxy/MXene adaptable network, which progressively dissociates with increasing temperature, leading to the formation of free ─C─O and –OH groups and creating additional sites for H‐bond interaction. Furthermore, the disruption of the H‐bond is primarily observed within the temperature range of 25–50 °C, indicating that the chemical bond activity is high at room temperature, resulting in the formation of a significant number of H‐bonds. Consequently, the free ─C─O and ─OH groups can interact and reorganize to form H‐bonds at room temperature, thereby imparting self‐healing capability to the epoxy/MXene adaptable network under ambient conditions.

A 2D correlation spectroscopy (2DCOS) analysis of USEP‐M_0.5_ was performed to extract additional spectral information and confirm the thermal reaction order of the different groups in the H‐bond. Based on Noda's rule^[^ ^34^ ^]^ and the cross peaks symbols observed in the synchronous and asynchronous 2DCOS (Figure 1e,f; Table S1, Supporting Information), the thermal reaction order of the different groups was determined as follows: 1701.5 < 3460.4 < 3325.9 < 1716.6 cm^−1^. These findings indicate that the H‐bonds bonded in ─C═O moiety dissociates at lower temperatures than those bonded ─N─H moiety. Consequently, the ─C═O moiety exhibits greater mobility and a higher temperature sensitivity within the H‐bond framework compared to the ─N═H moiety, as evidenced in previous studies.^[^ ^35^ ^]^

Density functional theory (DFT) simulations are further implemented to understand the interaction mechanism of interfacial non‐covalent hydrogen bonds before and after modification of MXene nanosheets. The bonding modes of H‐bonds and binding energies at the interface between MXene and USEP (MXene/USEP), MXene and DA (MXene/DA), DA and USEP (DA/USEP), and UPy and USEP (UPy/USEP) are shown in Figure 1g,h, respectively. For simplicity, side‐chain UPy units are used to represent USEP molecular chain for the simulations. The binding energy of A/B system (Δ*E*_A/B_) can be calculated according to Equation 1 below:

(1) $$\Delta E_{A/B}=E_{\mathrm{total}}-E_{A}-E_{B}$$

where *E*_total_ is the total energy of the system, *E*_A_ and *E*_B_ represent the energy of substances A and B, respectively. Apparently, after UPy modifications, Δ*E* decreased from −1.72 eV (Δ*E*_MXene/EP_) to −2.23 eV (Δ*E*_UPy/USEP_), revealing stronger binding between USEP and MXene/UPy. This is attributed to the robust quadruple H‐bond formation. For MXene/DA nanosheets, dopamine can polymerize in situ on the MXene nanosheets and release hydrogen (H_2_). Its binding energy (Δ*E*_MXene/DA_ = *E*_total_ – *E*_MXene_ – *E*_DA_ + *E*_H2_) is as low as −3.74 eV, demonstrating the effectiveness of dopamine‐modified MXene nanosheet strategy. However, the binding energy (Δ*E*_DA/EP_ = −1.43 eV) is higher than that of Δ*E*_MXene/EP_ due to the weaker H‐bond between the dopamine and USEP molecular chains. Therefore, the MXene/UPy nanosheets grafted with UPy are able to produce more robust physical binding sites with the USEP matrix, so that the nanocomposites have desirable mechanical and self‐healing properties.

### 2.3 Mechanical Properties of Epoxy/MXene Adaptable Network

**Figure 2:**
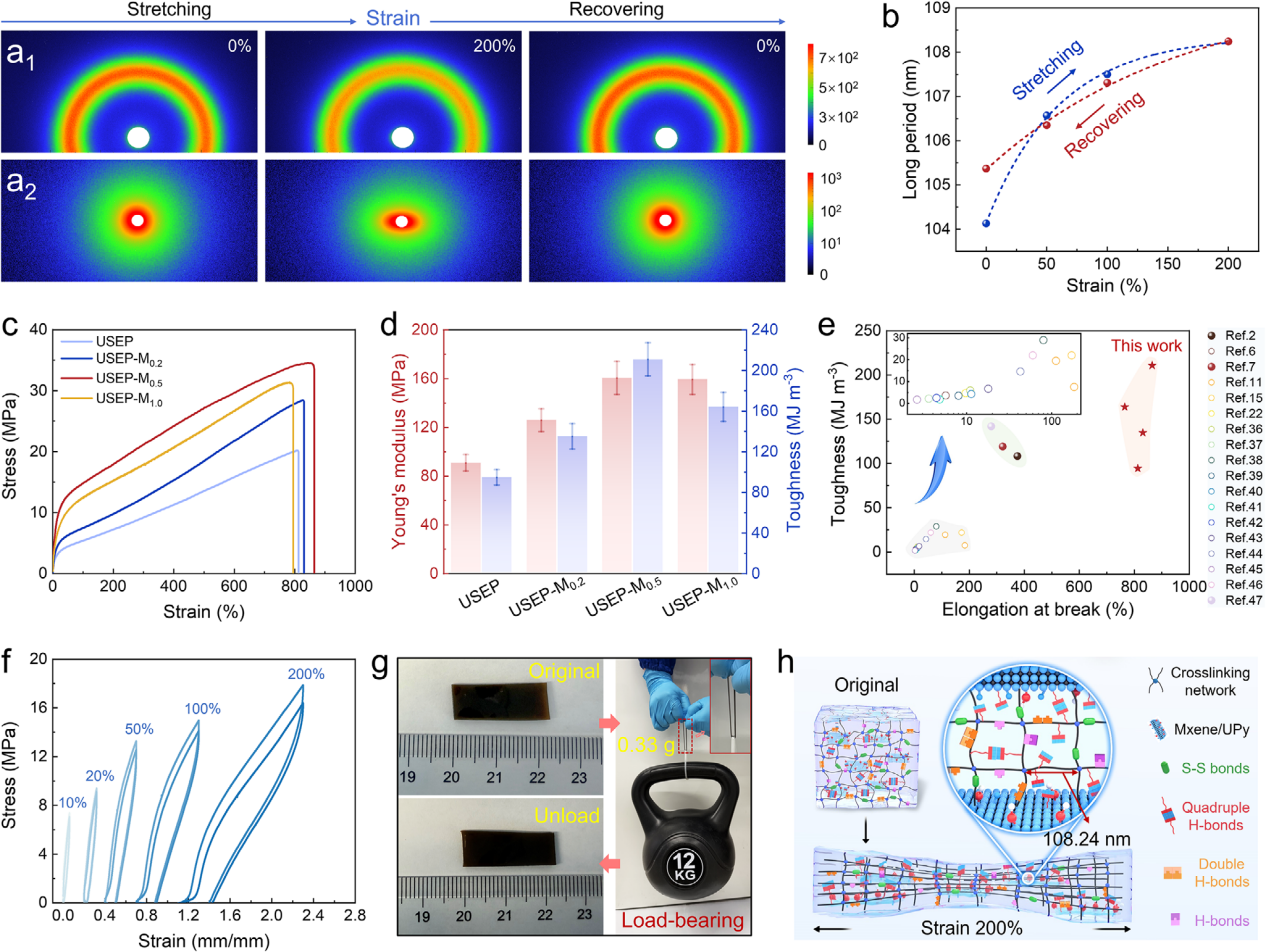
(a_1_) 2D WAXS patterns, (a_2_) 2D SAXS patterns, and b) long period values of USEP‐M_0.5_ at varying stretch–recovery strains. c) Stress–strain curves and d) Young's modulus and toughness values of epoxy. e) Comparison of the mechanical properties of the proposed material with those of toughened epoxy resins from the literature. f) Cyclic stress‐strain curves at different maximum strains of USEP‐M_0.5_. g) Load‐bearing optical images and h) schematic diagram of mechanical properties enhancement mechanism in USEP‐M_0.5_.

To assess the influence of multi‐type dynamic adaptable networks and IA‐nacre structures on the mechanical behavior of the epoxy resin, in situ wide/small angle X‐ray scattering (WAXS/SAXS) analyses were conducted on USEP‐M_0.5_ and USEP composites under various stretch‐recovery strains. The scattering pattern (**Figure** **2**a) shows that USEP‐M_0.5_ is isotropic and amorphous, yet phase‐separated. As the strain increases, the anisotropic 2D SAXS pattern reveals strain‐induced crystallization, characterized by a shift in the scattering signal from a uniform distribution to the equatorial direction. This change indicates that the isotropic MXene/UPy nanosheet structure aligns with the stress direction. This crystallization is attributed to the shift of the (002) plane of the soft segment (polytetramethylene ether glycol, PTMEG) from 18.8° to 19.5° during stretching (Figure S13, Supporting Information), which indicates the phase separation of the PTMEG soft phase. This separation leads to the creation of a temporary interface between the crystalline and amorphous regions, helping to resist external forces.

This specific microphase‐separated structure facilitates the formation of a robust, 3D physically cross‐linked network. This network enables dynamic bond breaking and reformation during the molecular chain movement, while simultaneously confining the epoxy molecular chain segments to promote strain‐induced microphase separation, ultimately enhancing the composite's tensile strength and toughness. In the case of USEP (Figure S14, Supporting Information), the diffraction signals in the 2D SAXS scattering pattern undergo a more pronounced change under 200% strain stretching. This indicates that the MXene/UPy nanosheets delay the crystallization of the composite coatings. This delay aids in the conformational adjustment of the molecular chains during stretching and also promotes phase separation based on the disentanglement of the molecular chains, significantly reducing the internal energy dissipation and thereby enhancing the material's toughness.

The scattering behavior of USEP‐M_0.5_ at varying levels of stretch‐recovery strains is shown in Figure S15 (Supporting Information). The long period value calculated using the Scheler equation (*d* = 2π/*q*) is ≈104.13 nm with its progression (Figure 2b). When the strain exceeds 100%, the strain‐induced crystallization restricts the rapid elongation of the PTMEG soft segment, resulting in pronounced strain‐hardening behavior and the formation of a more ordered periodic structure. Once the stress is relieved, the distance between the crystals gradually recuperates, demonstrating that the microphase separation structure of USEP‐M_0.5_ can effectively endure the matrix stress along the tensile direction. This behavior highlights its remarkable resilience, which not only enhances the material's strength but also imparts self‐healing capacities.

The stress‐strain curve of the epoxy/MXene adaptable network at a tensile rate of 50 mm/min is illustrated in Figure 2c. Representative test results are presented in Figure 2d and Table S2 (Supporting Information). The USEP samples exhibited an ultimate tensile strength (σ_b_) of 20.19 MPa and an elongation at break (*ε*) of 812.71%. In addition, Young's modulus (*E_Y_*) and toughness were measured at 90.52 MPa and 94.41 MJ m^−3^, respectively. The findings indicate that incorporating the side‐chain quadruple H‐bond and S‐S bonds significantly enhances the toughness and stretchability of the epoxy thermosets. The introduction on the IA‐nacre structure greatly enhances the strength and toughness of the epoxy/MXene adaptable network. At an MXene/UPy nanosheet content of 0.2 wt.%, the USEP‐M_0.2_ exhibits a σ_b_ value of 28.41 MPa and a toughness of 134.78 MJ m^−3^. The corresponding values of *ε* and *E_Y_* were augmented to 830.90% and 125.61 MPa, respectively.

Remarkably, the increased density of the quadruple H‐bond at the MXene/EP interface significantly boosts the toughness of USEP‐M_0.5_ to an impressive 210.75 MJ m^−3^, surpassing spider silk (160 MJ m^−3^) by a factor of 1.3. The σ_b_, *ε*, and *E_Y_* values reached 34.50 MPa, 864.72%, and 160.32 MPa, respectively, indicating excellent toughness and stretchability. This indicates that the bionic structure construction strategy is effective in enhancing the strength and toughness of the material. Figure S16 (Supporting Information) presents an optical image of the tensile testing. The proposed toughened epoxy resin exhibits superior properties to other toughened epoxy resins documented in the literature (Figure 2e; Table S3, Supporting Information).^[^ ^2^, ^6^, ^7^, ^11^, ^15^, ^22^, ^36^, ^37^, ^38^, ^39^, ^40^, ^41^, ^42^, ^43^, ^44^, ^45^, ^46^, ^47^ ^]^

USEP‐M_0.5_ demonstrates remarkable resilience, as evidenced by its cyclic stretch‐recovery curves following two repeated cycles at varying strains (Figure 2f). Even after undergoing the 200% strain cycle, the elastic recovery efficiency remained impressive at 84.51% (Figure S17, Supporting Information). Figure 2g illustrates the load‐bearing behavior of USEP‐M_0.5_. Weighing just 0.33 g, it can effortlessly lift a 12.0 kg dumbbell and swiftly return to its original length once unloaded, demonstrating exceptional toughness and elasticity. This can be attributed to the incorporation of a quadruple H‐bond at the MXene/epoxy interface and the design of the biomimetic structure. The 2D nanosheets contribute to improving the material's strength, while the interfacial H‐bonds serve as cross‐linking sites. These bonds can effectively increase the aggregation density and kinetic activity of the molecular chain H‐bonds, enabling the material to withstand tensile stresses, facilitate rapid energy dissipation (measured as the ratio of hysteresis loop area to loading curve area), and ultimately enhance the material's toughness. As the proportion of the rigid nanosheets increased, they hindered the formation of the epoxy covalent network and the move of the chain segments, resulting in a decrease in the USEP‐M_1.0_ material's σ_b_ values and toughness to 31.41 MPa and 163.85 MJ m^−3^, respectively. The *E_Y_* and *ε* values dropped to 159.14 MPa and 795.29%, respectively.

In order to reveal the toughness enhancement mechanism of the epoxy/MXene adaptable network, we observed the SEM cross‐sectional microstructure of the USEP‐M_0.5_ before and after the tensile test. First, cut an unstretched USEP‐M_0.5_ sample and stretched another USEP‐M_0.5_ sample to fracture, then fixed the morphology with liquid nitrogen and analyzed its cross‐section by SEM. The surface of the cut sample (unstretched) is smooth and free of any visible voids or defects (Figure S18a,c, Supporting Information). After stretching, the fracture surface perpendicular to the stretching direction shows circular voids at the nanoscale (Figure S18b, Supporting Information). By comparison, in the transverse morphology, there are many obvious fracture lines at the end of nanosheets (Figure S18d, Supporting Information). It is the formation of these uniformly dispersed micro‐cracks that promotes stress dissipation, resulting in ultra‐high toughness.

The mechanism for the mechanical property improvement of the epoxy/MXene adaptable network is shown (Figure 2h). When the epoxy matrix is subjected to stress, the bionic inverse‐artificial nacre structure resists large deformations preventing the material from fracturing. At the same time, dynamic chemical bonds begin to break and absorb energy, enabling rapid dissipation of external energy. After the external force disappears, the broken chemical bonds reconstruction, restoring the initial state of the material. Consequently, the abundance of S‐S bonds, side‐chain quadruple and interfacial H‐bonds within the molecular chain forms a network of strong, reversible cross‐links and sacrificial bonds. This network is crucial for promoting dynamic bond activity and enabling substantial energy dissipation. Furthermore, the micro‐phase separation structure formed by the multi‐type chemical bond adaptable network and IA‐nacre structure enables the chemical bonds to reduplicative breaking and reconstruction during molecular chain slip, resulting in enhanced strength and toughness for the composite. Consequently, the challenge of balancing high tensile strength with excellent toughness in thermosetting resin systems is effectively addressed.

### 2.4 Epoxy/MXene Adaptable Network Room/Low‐Temperature Self‐Healing Behavior

**Figure 3:**
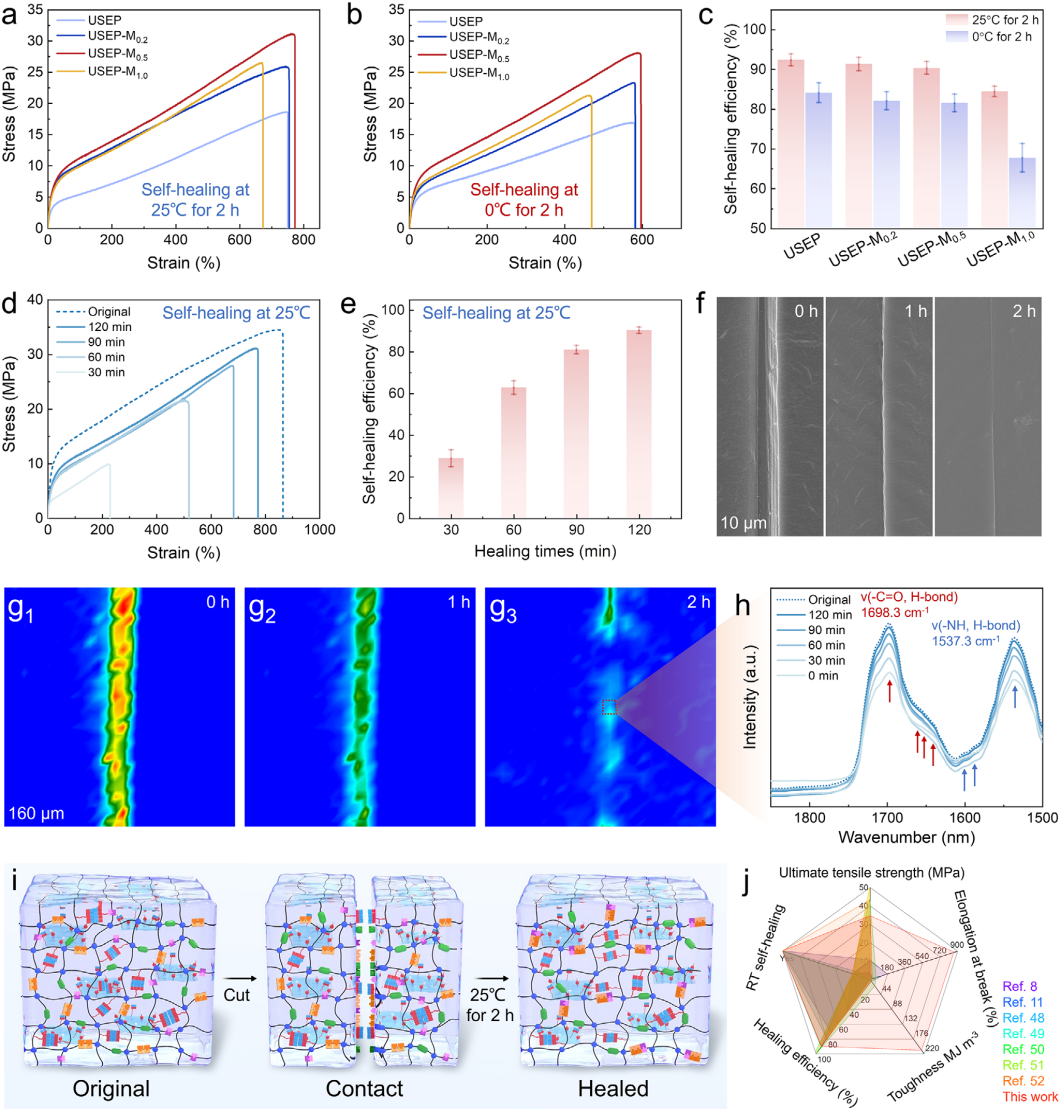
Stress–strain curves of epoxy after self‐healing under various conditions: a) 25 °C for 2 h and b) 0 °C for 2 h. c) *η* values of epoxy under different conditions. d) Stress–strain curves, e) *η* values, (f) SEM images, g) micro‐IR imaging images, and h) IR spectra of USEP‐M_0.5_ at different healing times. i) Illustrations of the self‐healing mechanism of USEP‐M_0.5_. j) Comparison of the mechanical properties and self‐healing capabilities of the proposed USEP‐M_0.5_ with those of self‐healing epoxies reported in the literature.

The enhancement of the dynamic chemical bond movement activity facilitates the rapid reconstruction of the broken chemical bonds, demonstrating an efficient damage repair capability. The self‐healing capacity of the composites’ mechanical properties was assessed at ambient temperatures (25 °C) and ≈0 °C using tensile testing. The effect of different MXene/UPy nanosheet doping levels was investigated. The stress‐strain curves are shown in **Figure** **3**a,b, with the corresponding test results listed in Table S4 (Supporting Information). Once the sample is cut in half, its mechanical strength can be regained by bringing the halves into contact at room or low temperature and allowing them to rest for 2 h. The self‐healing efficiency (*η*) is defined as the ratio of the restored σ_b_ value to the original of the material. The σ_b_ and toughness of the epoxy/MXene adaptable network initially increased and then decreased with increasing nanosheet content following self‐healing at both room and low temperatures. The synergistic interplay of multi‐type chemical bonds and bionic structures within USEP‐M_0.5_ results in superior mechanical performance. Notably, *η* values at room and low temperatures reached 90.4% and 81.5%, respectively (Figure 3c).

Nevertheless, the 2D nanosheets limit the reformation of the disrupted chemical bonds and restrict the mobility of the molecular chains, leading to a gradual decline in *η* values as the nanosheet content increases. The most rapid decrease occurred at an MXene/UPy content of 1 wt.%, where the *η* values dropped to 84.4% at room temperature and 67.7% at low temperature. Due to the combined influence of the high‐density interfacial H‐bond and the biomimetic structure, the *η* values of USEP‐M_0.2_ and USEP‐M_0.5_ exhibited minimal changes. Consequently, USEP achieved the highest *η* values without any external energy assistance, reaching 92.4% at room temperature and 84.1% at low temperatures. Notably, the variation in *η* values across different temperatures results from the decreased mobility of the molecular chains at lower temperatures. Furthermore, the *T_g_* value of the epoxy covalent network, ranging from 0 to 25 °C, is a key factor contributing to the reduction in *η* values at low temperatures. Conversely, the *T_g_* value of the multi‐type chemical bond network is less than −70 °C, allowing for the swift reformation of disrupted chemical bonds at both low and room temperatures, thereby exhibiting excellent self‐healing capabilities.

To assess the repair stability of the epoxy/MXene adaptable network at ambient temperature, we conducted five cycles “damage/healing” behavior study on USEP‐M_0.5_. The sample was self‐healing at room temperature for 2 h immediately after fracture during the tensile test, and then the tensile test was repeated. Typical stress‐strain curves and mechanical properties are shown in Figure S19 and Table S5 (Supporting Information), respectively. Obviously, even after repeating the “damage/healing” process, the σ_b_ value and toughness of the USEP‐M_0.5_ are still as high as 31.12 MPa and 157.72 MJ m^−3^ after 5 cycles, respectively, and the mechanical strength self‐healing efficiency is 90.2%, showing excellent repair stability.

The stress‐strain curves of USEP‐M_0.5_ at different self‐healing times under ambient conditions are shown in Figure 3d, with the corresponding test results provided in Figure 3e and Table S4 (Supporting Information). The *η* values of the samples cut into two pieces after contact for 30, 60, and 90 min were 28.8%, 62.8%, and 81.0%, respectively, while the corresponding σ_b_ values were 9.94, 21.66, and 27.96 MPa. The results show that the material's self‐healing process primarily occurs in the early and middle stages. During the initial phase, the damaged samples gradually come into contact due to their elasticity. Subsequently, a significant number of the broken chemical bonds undergo exchange and reconstruction, facilitating self‐healing. Due to the swift reformation of the dynamic chemical bonds, the tensile strength of USEP‐M_0.5_ can recover over 80% after 90 min of self‐healing at room‐temperature, showing exceptional self‐healing performance at room temperature. This was confirmed by the SEM images of the USEP‐M_0.5_ material surface during the defect self‐healing process (Figure 3f). After an hour of self‐healing at ambient temperature, the size and extent of the defects progressively diminished. After 2 h, the only remaining sign of damage was the interface where the material had been broken, with the defects almost imperceptible. Moreover, the healed sample exhibited a restored elongation exceeding 600%, as shown by the optical images of the self‐healing process (Figure S20, Supporting Information).

To elucidate the self‐healing mechanism of the epoxy/MXene adaptable network, the reformation process of the disrupted chemical bonds in the molecular chains was observed in situ at the microscopic scale using micro‐IR spectroscopy. The micro‐IR images and spectra of USEP‐M_0.5_ at various self‐healing times are shown in Figure 3g,h, respectively. Upon the initial contact of the two sample pieces at room temperature, the H‐bonding intensity within the UPy, urethane, and urea groups, observed in the 1500–1800 cm^−1^ spectral range, was notably weak. This is shown by the prominent red‐yellow grooves in the micro‐IR images. With extended contact time, the broken chemical bonds are progressively reformed. This is indicated by a gradual increase in the H‐bond intensity at the defects, while the defect width in the micro‐IR images decreases and turns light green. The recovery pattern of the broken H‐bond intensity mirrors that of the material's mechanical properties, with both exhibiting the fastest repair rate during the early and middle stages. After 2 h of contact at ambient temperature, most of the disrupted H‐bonds at the defects were reformed, with the intensity of H‐bond nearly matching that of the original intact coating surface. The color of the defects in the micro‐IR images shifted predominantly to light blue or even blue. The healing efficiency of the H‐bond intensity was estimated to be ≈91.9% after 2 h of self‐healing, demonstrating remarkable room‐temperature self‐healing capability.

The exceptional self‐healing capability exhibited by USEP‐M_0.5_ can be primarily ascribed to the multiple chemical bond types inherent within the adaptable network. A schematic representation of its self‐healing mechanism is provided in Figure 3i. In the event of material damage, the material initially relies on its elasticity to facilitate gradual contact between the damaged surface. Subsequently, the multi‐type chemical bond network, rich in H‐bond/S‐S bond acceptors and donors, allows for the swift exchange and reformation of the ruptured H‐bond/S‐S bonds, aiding in the restoration of the material's original properties. In addition, the low *T_g_* value (less than −70 °C) of the multi‐type chemical bond network ensures that the material remains mobile at both room and low temperatures. Consequently, the USEP‐M_0.5_ adaptable networks exhibited excellent self‐healing properties and toughness. Based on the current understanding, this epoxy exhibits the highest toughness and elongation among all the self‐healing epoxies reported in the literature to date (Figure 3j; Table S6, Supporting Information).^[^ ^8^, ^11^, ^48^, ^49^, ^50^, ^51^, ^52^ ^]^ The bionic multi‐type dynamic adaptable network developed through this hierarchical assembly method effectively addresses the trade‐off between good mechanical strength, high toughness, and rapid room‐temperature self‐healing capability, offering valuable insight for designing ultra‐strong and tough self‐healing materials.

### 2.5 Underwater/Low‐Temperature Self‐Healing Anti‐Corrosion Behavior

**Figure 4:**
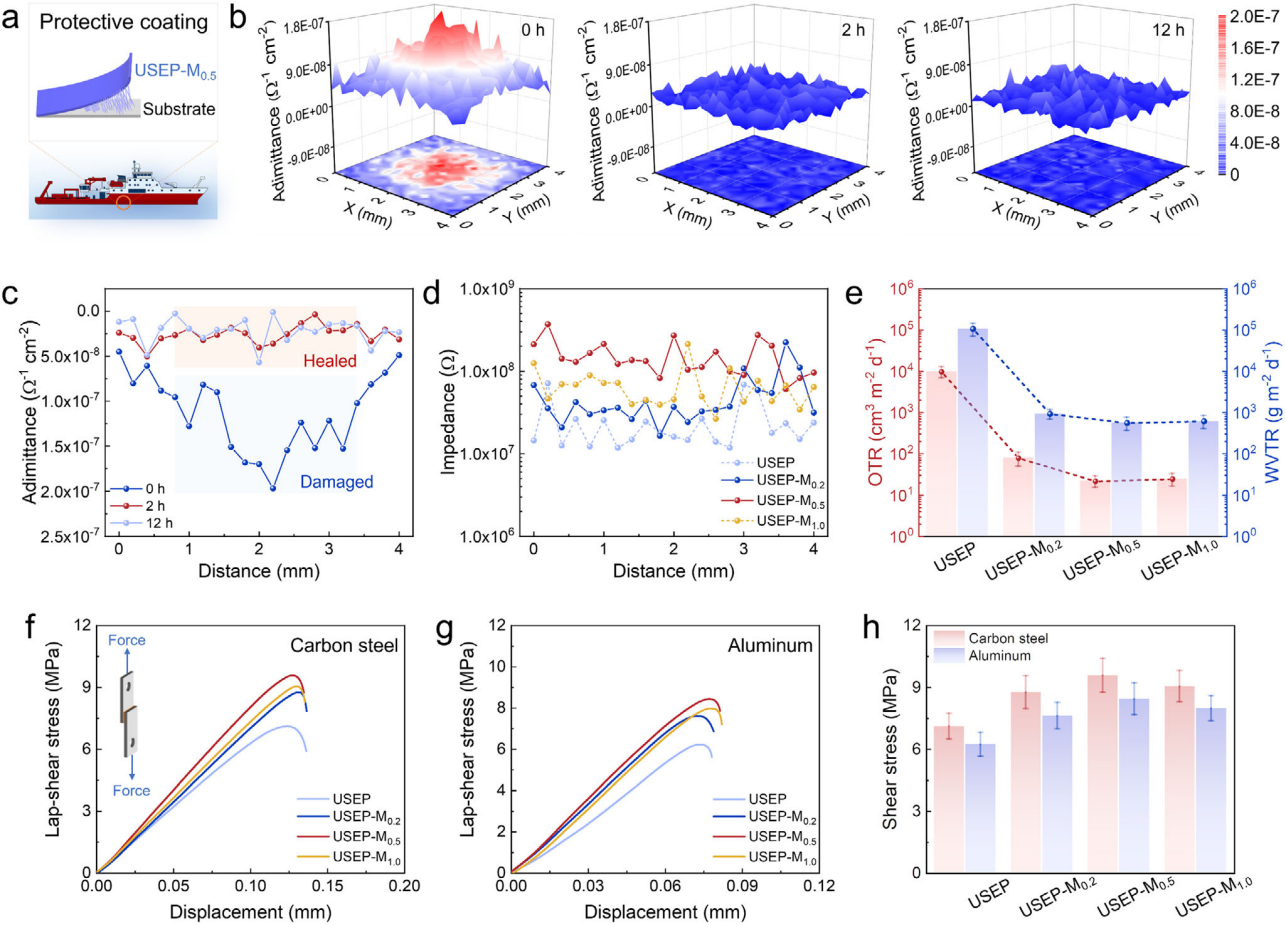
a) Structures and applications of the protective coating. b) LEIS images and c) admittance values of USEP‐M_0.5_ at various self‐healing times. d) Relationship between the impedance value and the distance in the epoxy. e) OTR and WVTR values of USEP‐M_0.5_. Stress–shear strain curves of the epoxy bonded to different metal substrates: f) Q235 carbon steel and g) aluminum. h) The shear strength of epoxy bonds to Q235 carbon steel and aluminum.

Leveraging the combined effect of reversible multi‐type dynamic adaptable networks and a bionic IA‐nacre structure, the proposed self‐healing material demonstrates the ability to restore its anti‐corrosion functionality in ice saltwater (3.5 wt.% NaCl, 0 °C). **Figure** **4**b shows the local electrochemical impedance spectroscopy (LEIS) test images of the USEP‐M_0.5_ coatings applied to the carbon steel surfaces (Figure 4a) after varying the immersion durations in ice saltwater. The introduction of scratches to the coating surface (at 0 h) results in a substantial increase in the admittance value (the inverse of the impedance) at the defect site. This phenomenon is visually manifested by a prominent red hue within the LEIS test image. After 2 h of self‐healing in ice saltwater, the defects were restored, exhibiting a blue color matching that of the intact coating surface. As the immersion time was extended to 12 h, the damage‐healed coating remained intact, demonstrating excellent stability in its anti‐corrosion performance. Figure 4c provides a visual representation of the admittance value recovery at the defect's deepest point along the *X*‐direction for varying immersion durations. Due to the protective influence of the hydrophobic chain segments and epoxy covalent networks on the H‐bonds and S‐S bonds, the USEP‐M_0.5_ coating demonstrates water insensitivity, enabling rapid self‐healing across various environments, including water, low, and room temperatures.

Figure 4d shows the impedance values of the composite coating in saltwater. As the MXene/UPy nanosheet content increases, the impedance of the coating initially increases and then decreases. The highest corrosion resistance was observed at 0.5 wt.%, with an impedance value of ≈1.32×10^8^ ohm. This can be attributed to the exceptional impermeability of the MXene/UPy nanosheets, which significantly improves the coating's ability to prevent the penetration of corrosive substances. However, the excessive addition of nanosheets can lead to interface compatibility issues with the epoxy matrix, reducing the impermeability and corrosion resistance. This is supported by the gas permeability analysis of the composite (Figure 4e; Table S7, Supporting Information). The addition of parallel consistent MXene/UPy nanosheets significantly reduced the oxygen transmission rates (OTR) and water vapor transmission rates (WVTR) of the epoxy coatings, achieving the optimal barrier performance at a 0.5 wt.% concentration. The OTR and WVTR values of USEP‐M_0.5_ were 21.7 cm^3^ m^−2^ d^−1^ and 554.2 g m^−2^ d^−1^, respectively. These values are two orders of magnitude lower than those of the USEP, highlighting its exceptional gas impermeability. However, the gas permeability of USEP‐M_1.0_ increased slightly because of the decreased interfacial compatibility. Therefore, the USEP‐M_0.5_ coating provides the best corrosion protection, with its protection mechanism shown in Figure S21 (Supporting Information).

The polyhydroxy structure within the epoxy/MXene adaptable network enhances the composite coating's interfacial bonding properties, thanks to the biomimetic design inspired by the mussel byssus. The stress‐shear strain curves of the four types of epoxy resins between two metal plates were analyzed using the lap‐shear method, and the changes in the bonding strength were positively related to the alterations in the materials’ mechanical properties, as shown in Figure 4f–h and Table S8 (Supporting Information). The bonding strength of USEP‐M_0.5_ on the Q235 carbon steel and aluminum alloy surfaces reached 9.58 and 8.44 MPa, respectively. In addition, the epoxy/MXene adaptable network demonstrating excellent water resistance and chemical stability (Figures S22 and S23, Supporting Information). As a result, the USEP‐M_0.5_ coatings, with their high strength, ultra‐toughness, impermeability, robust interface bonding, and multiple environmental self‐healing properties, are believed to have significant application potential for corrosion protection in complex marine environments.

### 2.6 Stable Underwater Flexible Sensing Behavior

**Figure 5:**
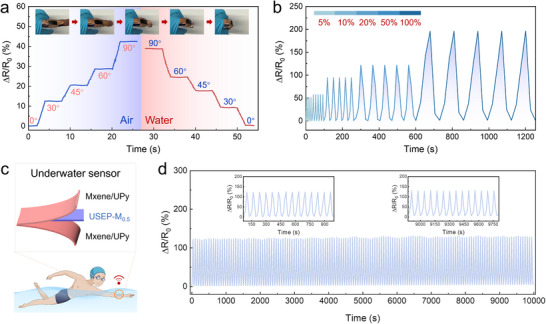
a) Relationship between ∆R/R0 and the bending angle in both air and water. b) Variation of ∆*R/R_0_* at different maximum strains. c) Construction of the sensor and its underwater applications. d) Sensor stability under 50% strain over a 10 000 s period.

Leveraging the high conductivity of the MXene/UPy nanosheets and the exceptional tensile strength and water‐insensitive properties of the USEP‐M_0.5_ coating, an underwater MXene/UPy–USEP‐M_0.5_–MXene/UPy (M/EP/M) sensor was developed for real‐time monitoring of human joint motion. The structure of the sensor is shown in **Figure** **5**c. The distinctive interconnected conductive network design of the resulting material facilitated the rapid resistance change (*∆R/R_0_*) sensing behavior. Figure 5a illustrates the M/EP/M sensor attached to a finger, enabling bending movement in both air and underwater environments. As the finger flexed, the resistance increased in direct proportion to the bending angle. Similarly, the sensor demonstrates stable behavior in an aqueous solution, where the resistance value decreases and then returns to its initial state as the finger is released after being immersed in water. This observation indicates that the signal feedback is driven by migration within the conductive network of M/EP/M, rather than by ion movement in the water. No matter in air or water, the relative resistance changes and bending angle of the M/EP/M sensor are linearly related (correlation coefficient *r* > 0.99), and the sensitivity (*S*) values are 0.477 and 0.434, respectively, indicating that the sensor has high response ability and good stability (Figure S24, Supporting Information).

The stability of the M/EP/M sensor was assessed by recording its resistance variations in situ during cyclic stretching‐recovery at strains from 5% to 100%, as shown in Figure 5b. A correlation between the strain and resistance increase was observed, with substantial reversibility upon strain recovery. This behavior is attributed to the strain‐induced narrowing of the ion migration channels and elongation of the migration pathways within the conductive network. Notably, the M/EP/M sensor provides a resistance signal with exceptional stability and reliability. Upon repeated stretching to 50% strain for 154 cycles, its ∆*R/R_0_* remained consistent (Figure 5d). Therefore, M/EP/M sensors hold great potential for applications in flexible sensing within complex marine environments.

## 3 Conclusion

Inspired by mussel nacre and byssus, a hierarchical assembly strategy is proposed for fabricating multi‐type dynamic chemically bonded epoxy/MXene adaptable networks. These networks exhibit ultra‐toughness and multi‐environmental self‐healing capabilities. Typically, the as‐prepared USEP‐M_0.5_ demonstrates remarkable toughness (210.75 MJ m^−3^), 1.3 times greater than that of spider silk, owing to the benefits of the flexible side chain quadruple H‐bonds and the bionic structure that regulates its mechanical properties. In addition, abundant dynamic bonds provide high‐density hydrogen and disulfide bond acceptors and donors, enabling effective energy dissipation. Meanwhile, due to the protection of dynamic bonds by hydrophobic chains and covalent crosslinked networks, USEP‐M_0.5_ shows rapid self‐healing capabilities across room‐temperature, low‐temperature, and aqueous environments. This discovery challenges the conventional understanding of thermosetting resins, establishing it as the toughest room‐temperature self‐healing epoxy resin to date. Furthermore, the bioinspired adaptable network imparts the composites with exceptional gas barrier properties, chemical resistance, and robust interfacial bonding, and the fabricated flexible sensor devices have excellent stability. It is believed that this elaborate hierarchical assembly strategy and the bionic epoxy/MXene adaptable network design offer a universal strategy for developing long‐term protective coatings and flexible sensors for complex marine environments.

## Conflict of Interest

The authors declare no conflict of interest.

## Supporting information



Supporting Information

## Data Availability

The data that support the findings of this study are available from the corresponding author upon reasonable request.
