# Supplementary material for: Engineering of Multi‐Dynamic Bonds Toward Room‐Temperature Self‐Healing Epoxy/MXene Adaptable Network with Record‐High Toughness
Source: Adv Sci (Weinh). 2025 Jun 23;12(33):e08780. doi: 10.1002/advs.202508780 (PMC12412468; doi:10.1002/advs.202508780)
Supplement: Supplementary file 1 — Supporting Information [file ADVS-12-e08780-s001.pdf]

ADVANCED SCIENCE
Open Access

Supporting Information

for *Adv. Sci.*, DOI 10.1002/advs.202508780

Engineering of Multi-Dynamic Bonds Toward Room-Temperature Self-Healing Epoxy/MXene Adaptable Network with Record-High Toughness

Xiaobo Zhu, Yu Hao, Liang-Feng Huang, Haichao Zhao and Liping Wang**

Supporting Information

Engineering of Multi-Dynamic Bonds toward Room-Temperature Self-Healing Epoxy/MXene Adaptable Network with Record-High Toughness

Xiaobo Zhu[1,2], *Yu Hao*[1,2], *Liang-Feng Huang*[1,2,3], *Haichao Zhao*[1,2,*], *and Liping Wang*[1,2,*]

[1] State Key Laboratory of Advanced Marine Materials, Ningbo Institute of Materials Technology and Engineering, Chinese Academy of Sciences, Ningbo 315201, China

[2] University of Chinese Academy of Sciences, Beijing 100049, China

[3] Research Center for Advanced Interdisciplinary Sciences, Ningbo Institute of Materials Technology and Engineering, Chinese Academy of Sciences, Ningbo 315201, China

[*] Corresponding author.

E-mail: zhaohaichao@nimte.ac.cn (H. Zhao); wangliping@nimte.ac.cn (L. Wang).

1. Methods

1.1. Materials

Ethyl acetoacetate (≥ 99%), dopamine hydrochloride (DA, ≥ 98%), and glycidol (≥ 97%) were purchased from Shanghai Macklin Biochemical Technology Co., Ltd.. Guanidine carbonate (≥ 99.5%), hexamethylene diisocyanate (HDI, ≥ 99%), 2-amino-2-methyl-1,3-propanediol (AMPD, ≥ 99%), ditin butyl dilaurate (≥ 95%), polytetramethylene ether glycol (Mn ~1000, PTMEG-1000), 4,4'-diaminodiphenyl disulfide (2S, ≥ 98%), isophorone diisocyanate (IPDI, ≥ 99%), and poly(propylene glycol) bis(2-aminopropyl ether) (Mn ~230, D230) were purchased from Shanghai Aladdin Biochemical Technology Co., Ltd.. Ethanol, tetrahydrofuran, acetone, N,N-dimethylformamide (DMF), ethyl ether, trichloromethane ($CHCl_3$), pentane and other solvents were purchased from Sinopharm Chemical Reagent Co., Ltd.. Few-layer $Ti_3C_2T_x$ MXene nanosheets were obtained from Newene Technology Ltd. with a conductivity of about 3500 S/cm. All reagents were used directly without treatment.

1.2. Synthesis of UPy and UPy-OH chain extenders

2-ureido-4[1H]-pyrimidinone (UPy) was prepared according to established methods in the literature.[1, 2] A reaction mixture containing 36 g of guanidine carbonate (20 mmol) and 57.28 g of ethyl acetoacetate (44 mmol) in 450 mL ethanol was refluxed for 12 h in a round-bottom flask under an inert atmosphere. After completion of the reaction, the mixture was filtered, washed three times with acetone, and then dried under vacuum at 60°C for 12 h to yield white 6-methylisocytosine (MIC). 10 g of MIC (80 mmol) and 80.64 g of HDI (480 mmol) were charged into a single-necked flask, and the reaction was refluxed at 100°C for 12 h under an N_2 atmosphere.

The reaction mixture was precipitated with 400 mL of pentane, resulting in the precipitation of the product. The precipitate was then isolated by filtration, washed thoroughly with acetone, and dried under vacuum at 50°C for 12 h to afford UPy as a white, air-stable powder.

To synthesize the UPy-OH chain extender, 11.72 g of UPy (40 mmol) and 6.3 g of AMPD (60 mmol) were combined, followed by the addition of 420 mL of $CHCl_3$. The mixture was then refluxed for 5 h under an N_2 atmosphere. Upon completion of the reaction, the solution turned a uniform milky white. It was then filtered and washed three times with $CHCl_3$. The solid was placed into a beaker, and 200 mL of DMF was added to ensure complete dissolution. The mixture was then subjected to centrifugation (9000 rpm, 10 min). The supernatant was collected, and 500 mL of ether was added to induce precipitation. In conclusion, the sediment was filtered and washed three times with acetone. The UPy-OH chain extender was obtained by vacuum drying at 60°C for 12 h.

1.3. Preparation of MXene/UPy nanosheets

To enhance the surface quality of the MXene nanosheets and introduce a variety of hydroxyl and amino functional groups. MXene/DA 2D nanosheets were synthesized through the oxidative polymerization of dopamine. Specifically, 0.8 g of MXene nanosheets were dispersed in 400 mL of Tris-buffer buffer (pH = 8.5) via sonication for 30 min. Subsequently, 0.4 g of dopamine was added and the mixture was stirred at 60°C for 24 h. The reaction mixture was centrifuged, and the product was washed three times with deionized water and DMF. Finally, the MXene/DA was collected. Quadruple H-bond modified MXene/UPy nanosheets were prepared by grafting UPy onto the dopamine surface through an addition reaction. Initially, 0.5 g of MXene/DA nanosheets

were dispersed in 80 mL of DMF. Subsequently, 0.1 g of UPy dissolved in 5 mL of DMF and 0.1 mL of DBTDL was added dropwise. The reaction was conducted under reflux at 100°C for 16 h. After completing the reaction, the product was filtered, washed three times with DMF, and collected to yield MXene/UPy nanosheets.

1.4. Epoxy/MXene adaptable network synthesis and design

Epoxy/MXene adaptable networks were prepared using a hierarchical assembly method to replicate the structure of the mussel nacre and byssus. Precisely, 96 g of PTMEG-1000 (96 mmol), 2.98 g of 2S (12 mmol), and 4.776 g of UPy-OH chain extender (12 mmol) were combined in a single-necked flask. Subsequently, 40 mL of DMF was introduced, and the reaction mixture was stirred under open conditions at 110°C for 30 min to ensure the complete removal of water from the system. Subsequently, 53.35 g of IPDI (240 mmol) was introduced under a N_2 atmosphere to synthesize a polyurethane (PU) prepolymer (M_n = 10280 g mol^{-1}), and the reaction proceeded at 80°C for 3 h. To obtain the epoxy (EP) prepolymer, 17.78 g of glycidol (240 mmol) was added to the reaction mixture after the initial reaction was completed. The reaction was conducted at 80°C for 3 h. To prepare the USEP dynamic epoxy networks containing quadruple H- and S-S bonds, 13.8 g of D230 (60 mmol) was added to the reaction mixture. The mixture was thoroughly stirred, poured into a mold, and cured at 60°C for 48 h.

For the USEP-M_x adaptable network, the MXene/UPy nanosheets (0.2 wt.%~1.0 wt.% relative to the USEP epoxy mass) were incorporated before the completion of the curing reaction. Once uniformly dispersed, D230 was added, and the parallel alignment of the MXene/UPy nanosheets within the resin was achieved through slow evaporation-induced assembly. This process involved

curing at 40°C and 50°C for 12 h each, followed by curing at 60°C for 30 h, resulting in the formation of a bioinspired inverse artificial nacre structure epoxy/MXene adaptable network.

1.5. M/EP/M sensor assembly

An independent MXene/UPy film was prepared by vacuum filtration of 6 mL of a 10%-MXene/UPy DMF dispersion. Subsequently, 3 mL of USEP-$M_{0.5}$ emulsion was introduced dropwise to the surface-dried film. After 16 h in the fume hood, an additional 6 mL of 10%-MXene/UPy DMF dispersion was added dropwise. In summary, after complete infiltration of MXene/UPy into the epoxy/MXene adaptable network, the MXene/UPy–USEP-$M_{0.5}$–MXene/UPy (M/EP/M) sensors were prepared by curing at 60°C for 12 h.

1.6. Characterization

The structural composition, morphology, and dimensions of the 2D nanosheets were analyzed using TEM (F200, JEOL) and SPM (Dimension ICON, Bruker). The cross-sectional and surface morphologies of the epoxy at various self-healing times were examined using SEM (S4800, Hitachi). The thermal analysis of the materials was performed using a differential scanning calorimeter (DSC214, Netzsch) and a thermogravimetric analyzer (TG209F1, Netzsch), with measurement ranges of -150°C to 100°C and 30°C to 800°C, respectively. Both analyses were conducted in an argon atmosphere with a heating/cooling rate of 10 °C/min. The structural information of the materials and in situ chemical bond changes during the self-healing process were observed using a micro-infrared spectrometer (Cary660+620, Agilent). The temperature-dependent IR spectra were collected using a NICOLET 6700 Fourier transform infrared spectrometer (Thermo Scientific), and the 2D COS maps were analyzed using 2D shige software.

2D small-angle X-ray scattering (Xeuss 3.0 UHR, XENOCS SAS) was employed to observe the structural changes of the materials under varying stretching and recovery strains in a vacuum environment.

1.7. Computational details

The density functional theory (DFT) calculations were performed within the VASP software.[3] The ion-electron interaction was described using the projector-augmented plane-wave[4] and the exchange-correlation interaction was described using the Perdew–Burke–Ernzerhof functional of the generalized gradient approximation.[5, 6] The single gamma-point grid sampling was used for Brillouin Zone integration. The vacuum layer larger than 15 Å was implemented to prevent the interaction between periodical structures. The zero-damping DFT-D3 functional[7] was used to describe the van der Waals (vdW) interactions. The cut-off energy was set as 450 eV, and all the systems were optimized until energy and force were less than 10^{-5} eV and 0.01 eV/Å.

1.8. Mechanical and self-healing tests

The mechanical and lap-shear properties of the samples were tested using universal material testing machines (Roell Z1.0 and Roell Z030, Zwick). The tensile specimens were developed in a standard dumbbell shape, with the tensile rate set at 50 mm min^{-1}. The lap-shear test was performed using epoxy resin as the adhesive, with Q235 carbon steel and aluminum alloy serving as the substrates. The bonding area was 10 × 15 mm^2. The self-healing properties of the material were assessed by cutting the sample into two sections and measuring the mechanical properties at various contact times under specific conditions. The self-healing efficiency (η) is defined as the ratio of the ultimate tensile strength to the original. The toughness is represented by the area under

the stress-strain curve, while Young's modulus is determined by the slope of the stress-strain curve during the elastic deformation phase. Each test was repeated five times to obtain the average values.

1.9. Anti-corrosion and gas permeability tests

The local electrochemical impedance spectroscopy (LEIS) of the materials in ice salt water (3.5 wt.% NaCl, approximately 0°C) was measured using a microelectrochemical workstation (VersaSCAN, Ametek), with a scanning area of 4 × 4 mm^2 and a frequency of 1 kHz. The oxygen and water vapor transmission rates of the materials were determined using a Labthink differential pressure gas permeation analyzer.

1.10. Sensing tests of M/EP/M sensors

The change in resistance ($\Delta R/R_0$) of the M/EP/M sensors was measured using a multimeter (Keithley DMM7510), with the samples secured using copper tape. The sensing test under varying strains was conducted using a combination of a universal material testing machine and a multimeter. Where R_0 represents the initial resistance of M/EP/M, and ΔR denotes the difference between the changed resistance and initial resistance.

Supplementary Figures

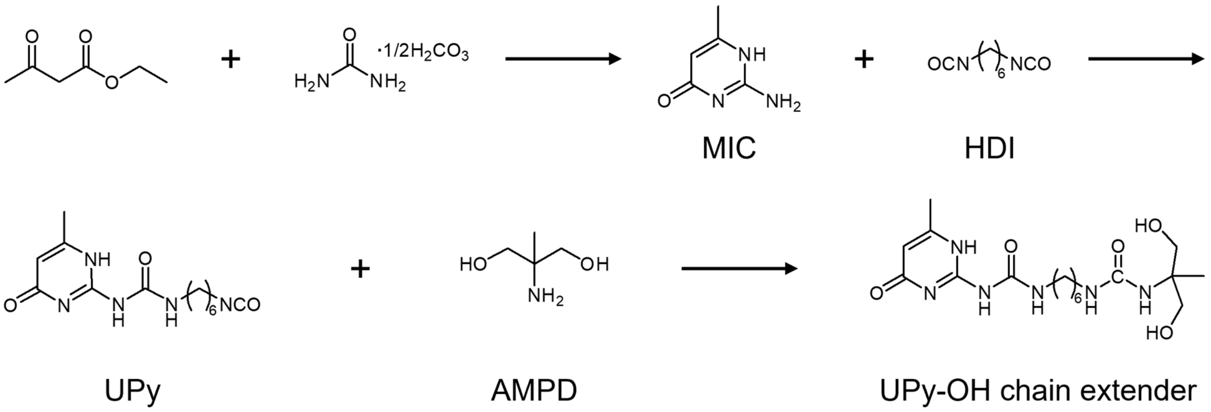

Fig. S1 Synthetic route of UPy and UPy-OH chain extender.

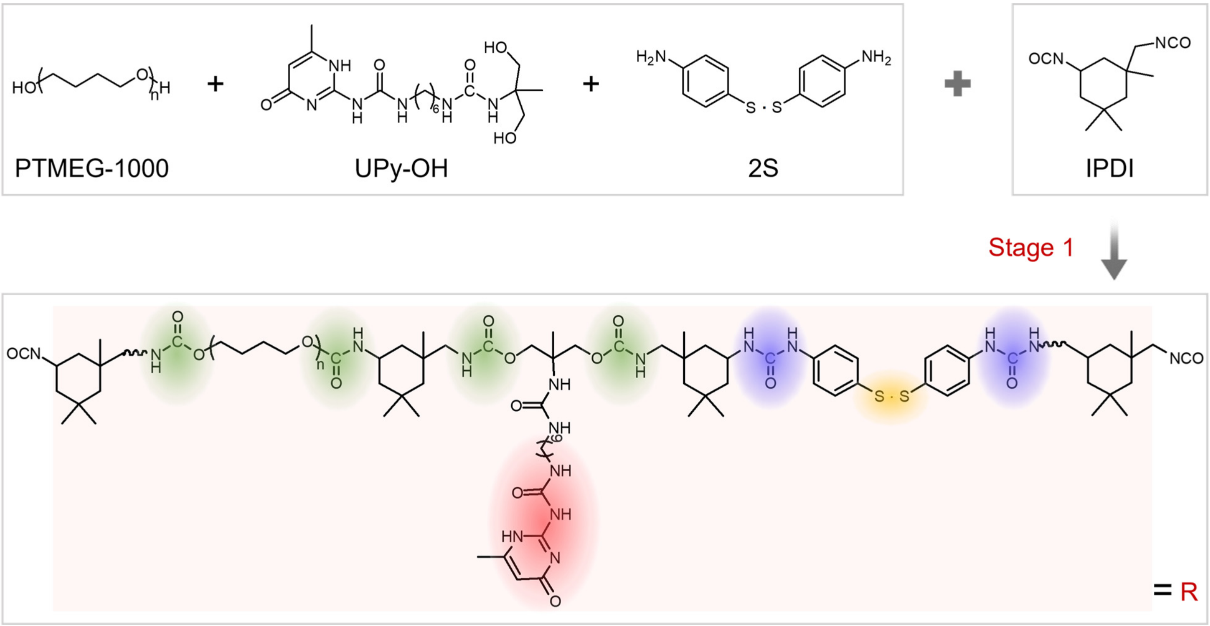

Fig. S2 Synthetic route of PU prepolymer.

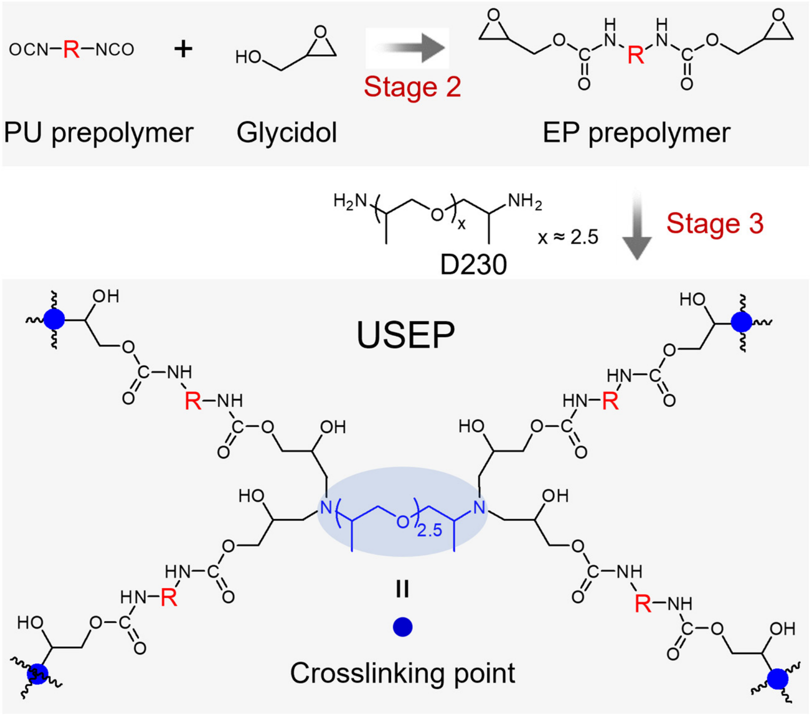

Fig. S3 Synthetic route of EP prepolymer and USEP.

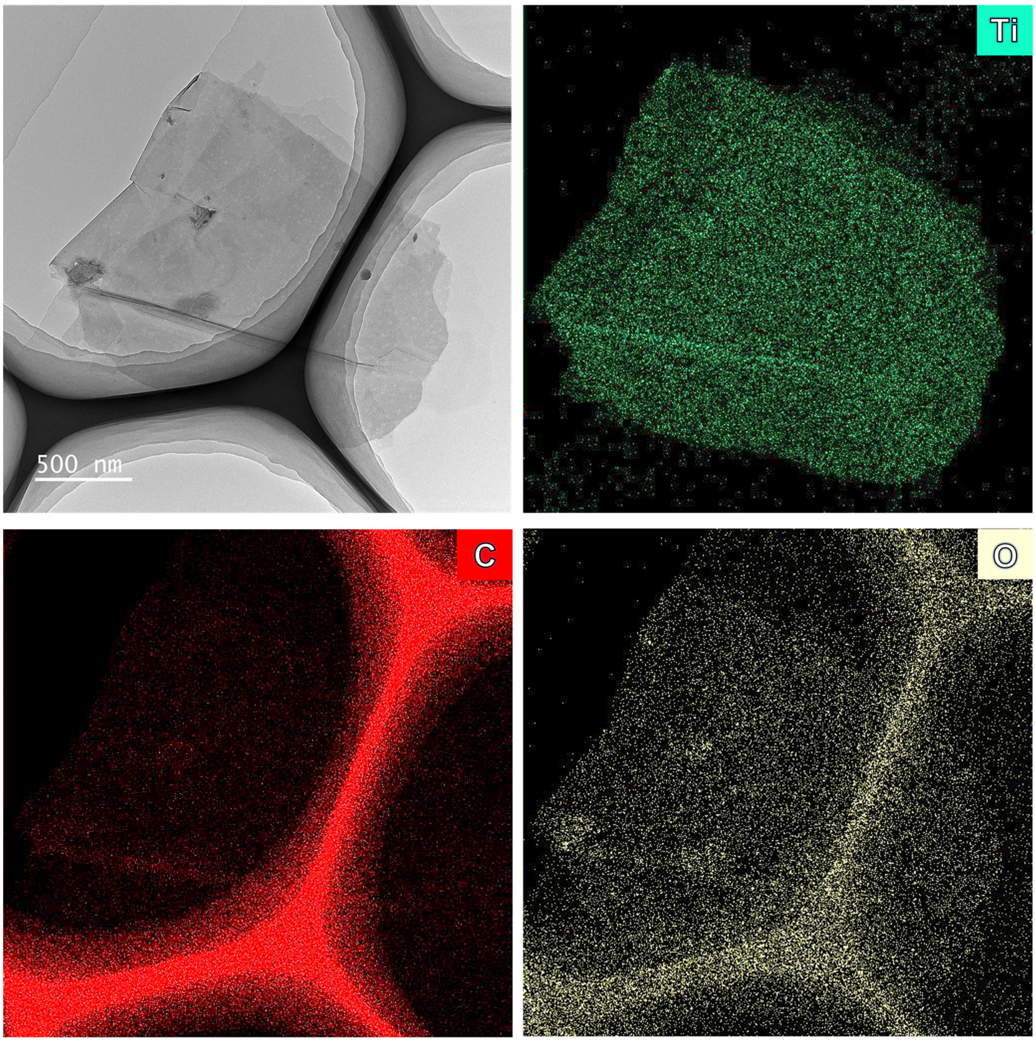

Fig. S4 TEM and TEM-EDS images of $Ti_3C_2T_x$ MXene nanosheet.

The elemental distribution shows that the $Ti_3C_2T_x$ MXene nanosheets predominantly consist of titanium (Ti) and oxygen (O) elements. However, after acid etching, the surface exhibited numerous residues and voids.

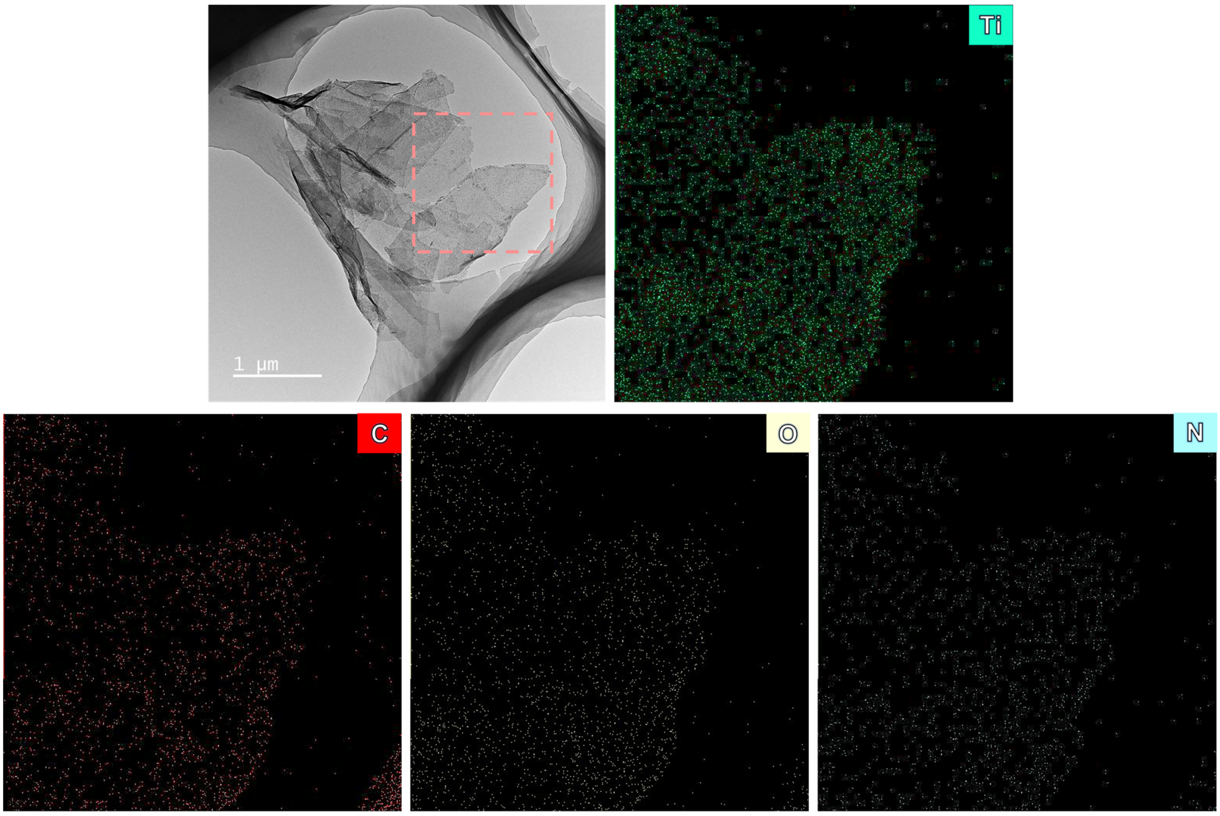

Fig. S5 TEM and TEM-EDS images of MXene/DA nanosheet.

In contrast to the initial state, the surface of the MXene/DA nanosheets after the dopamine (DA) coating exhibited a uniform coverage of fine particles, and all pores observed before the coating was filled. The elemental distribution map revealed a reduction in the Ti and O content, along with an increase in the carbon (C) and nitrogen (N) elements, confirming the successful modification with DA.

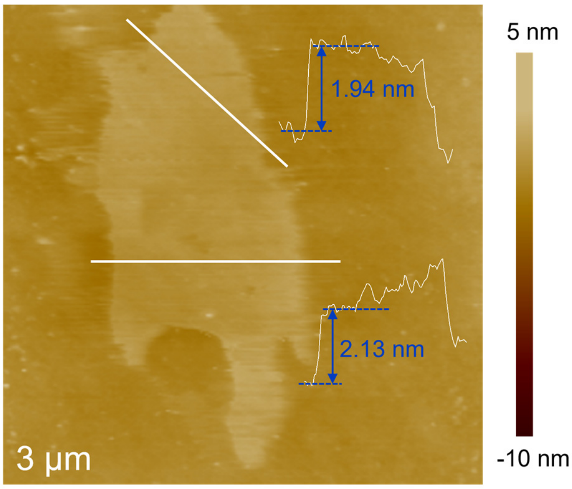

Fig. S6 SPM image of $Ti_3C_2T_x$ MXene nanosheet.

The scanning probe microscopy (SPM) analysis revealed that the nanosheet thickness was approximately 2.13 nm, indicating a significant length–to–thickness ratio of the nanosheet.

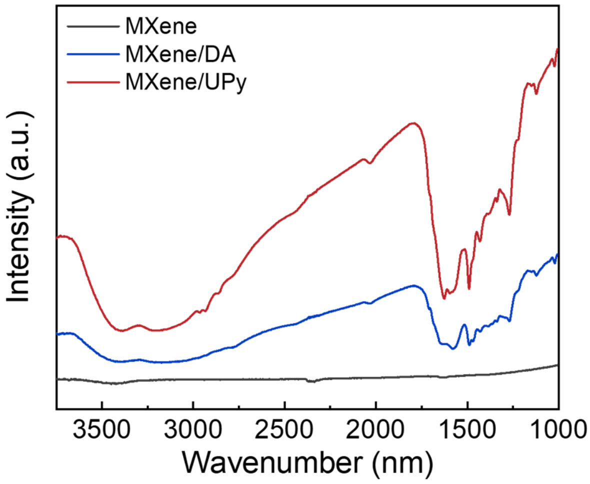

Fig. S7 FT-IR curves of nanosheets.

For the MXene/UPy nanosheets, the absence of –NCO group in the range of 2260–2280 cm^{-1} in the Fourier transform infrared (FT-IR) spectrum confirmed the successful grafting of UPy.

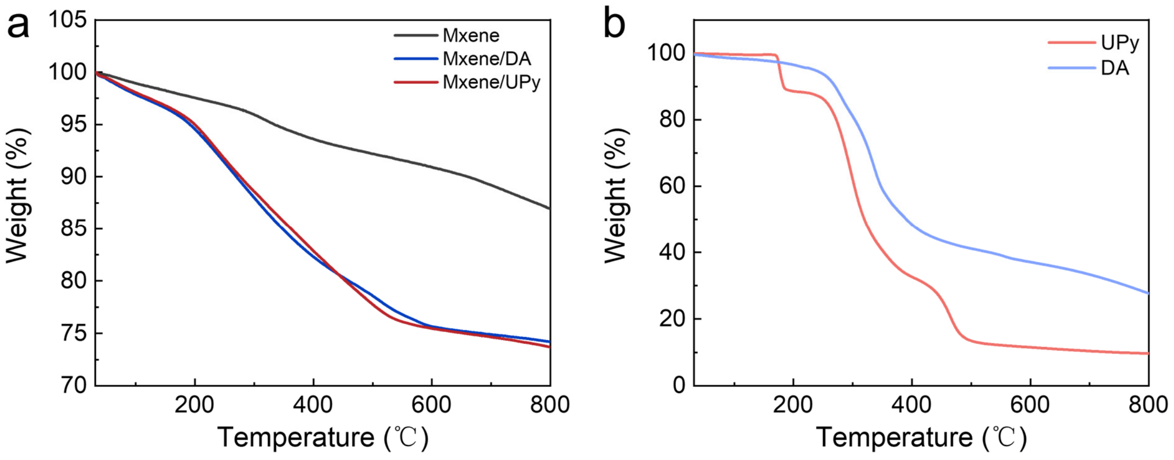

Fig. S8 TG curves of (a) nanosheets and (b) samples.

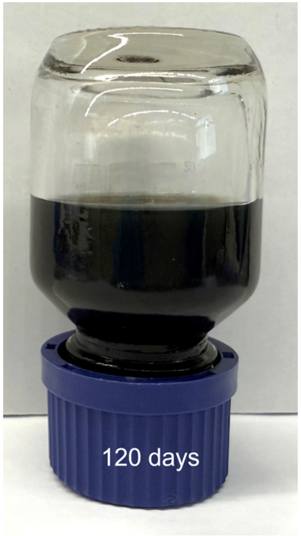

Fig. S9 Optical photograph of MXene/UPy nanosheets dispersed in deionized water for 120 days.

The nanosheets exhibited outstanding dispersion stability in aqueous solutions, as evidenced by the absence of sediment at the bottom of the sample bottle when inverted after 120 days at room temperature.

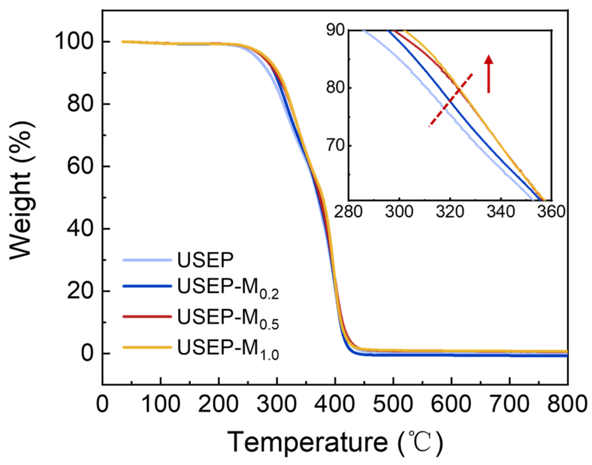

Fig. S10 TG curves of epoxy/MXene adaptable networks.

The TG curve data reveal that the four epoxy resins exhibit distinct decomposition temperature ranges for their hard and soft chain segments. The hard segments predominantly decompose between 220℃–360℃, whereas the soft segments degrade at higher temperatures, ranging from 360℃–450℃.

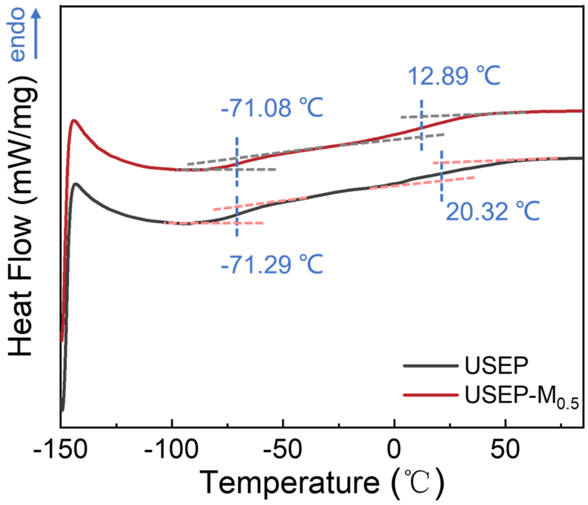

Fig. S11 DSC curves of USEP and USEP-$M_{0.5}$.

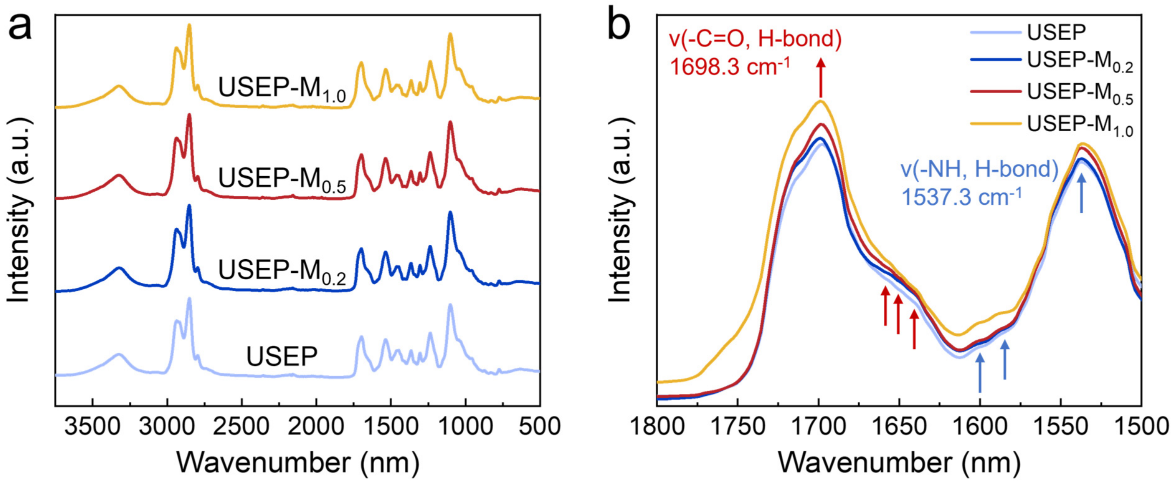

Fig. S12 Infrared spectra of epoxy/MXene adaptable networks: (a) 500–3500 cm^{-1} and (b) 1500–1800 cm^{-1}.

The structural compositions of the four epoxy resins were similar, with all exhibiting –N–H stretching (3323.9 cm^{-1}) and bending (1537.3 cm^{-1}) vibrations, –C–H vibrations (2750–3050 cm^{-1}), and the stretching vibration of –C=O in the H-bonds of UPy, urethane, and urea groups around 1698.3 and 1630–1670 cm^{-1}, respectively. Furthermore, the absence of the –NCO vibration peak in the 2260–2280 cm^{-1} range confirms the complete reaction of –NCO.

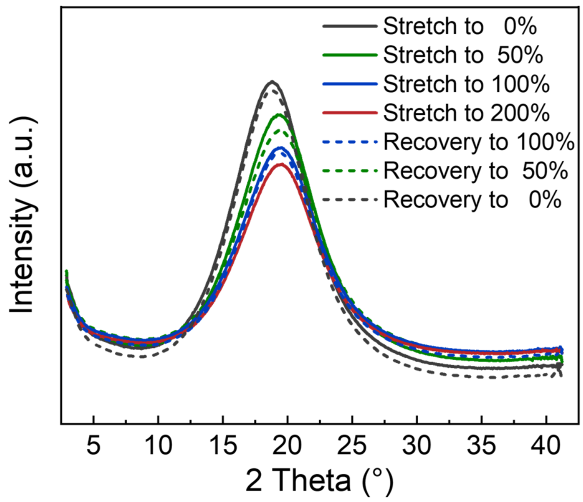

Fig. S13 1D WAXS integral curves of USEP-$M_{0.5}$ along equatorial direction at different stretch-recovery strains.

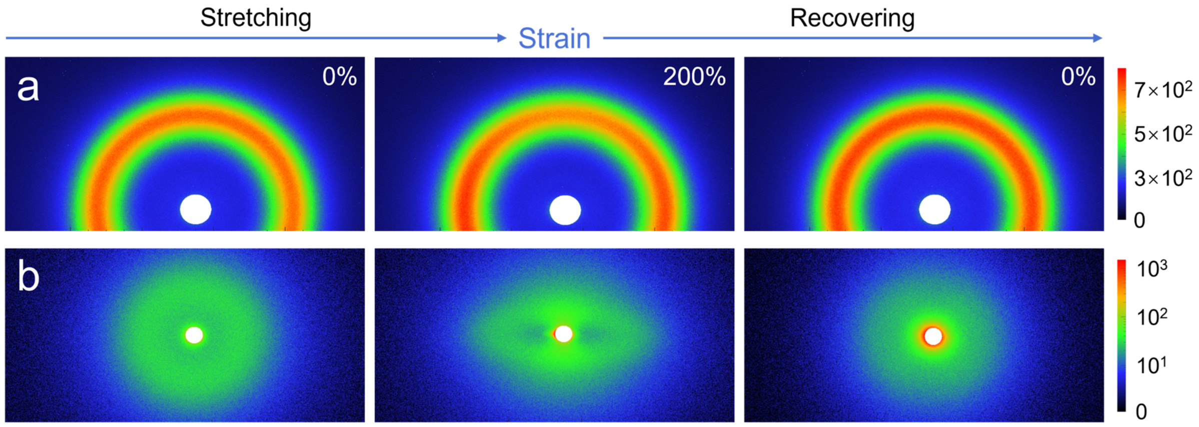

Fig. S14 (a) 2D WAXS and (b) 2D SAXS patterns of USEP-$M_{0.5}$ performed under varying stretch-recovery strains.

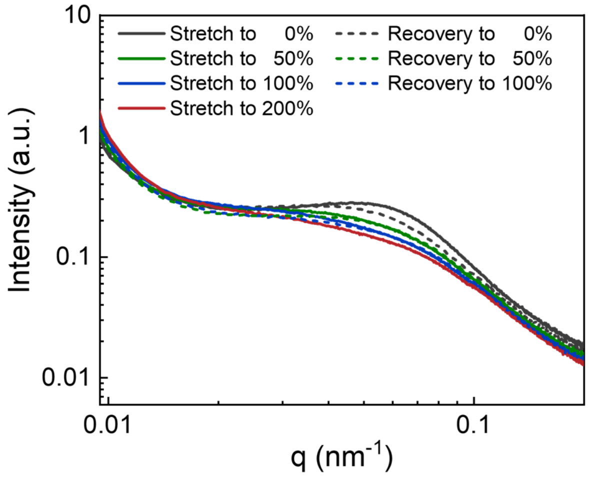

Fig. S15 Scattering curves of USEP-$M_{0.5}$ at varying stretch-recovery strains.

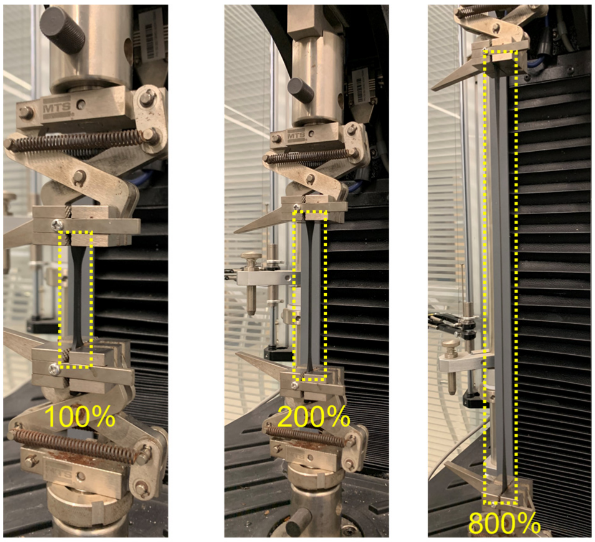

Fig. S16 Optical images of USEP-$M_{0.5}$ during tensile testing.

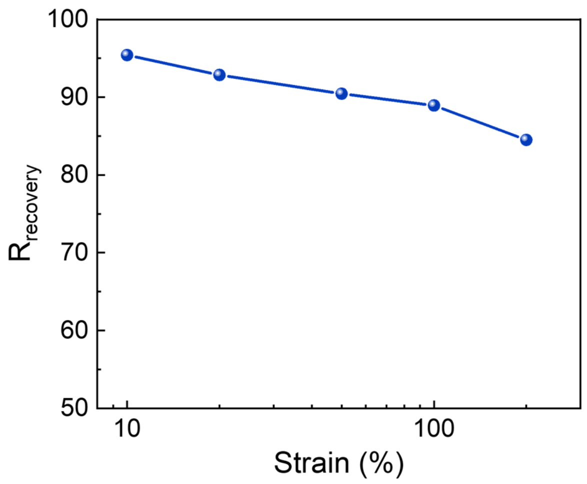

Fig. S17 Elastic recovery efficiency of USEP-$M_{0.5}$ under varying strains.

Elastic recovery efficiency ($R_{recovery}$), defined as the ratio of the area under the tensile loading curve of the second cycle to the first cycle.

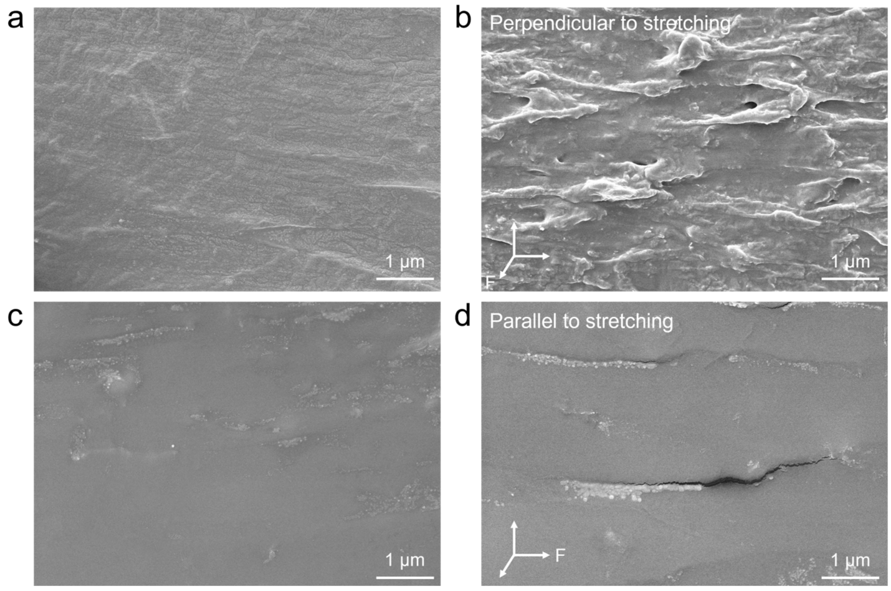

Fig. S18 SEM cross-sectional images of the USEP-$M_{0.5}$: (a, c) before tensile and (b, d) after tensile fracture.

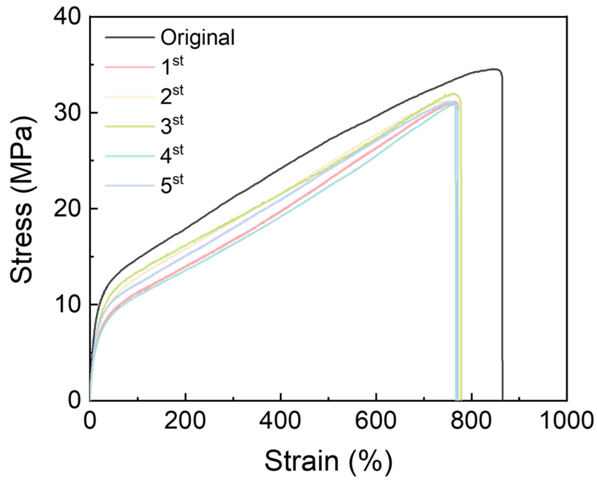

Fig. S19 Stress-strain curves of USEP-$M_{0.5}$ repaired at 25°C for 2 h with different "damage/healing" cycles.

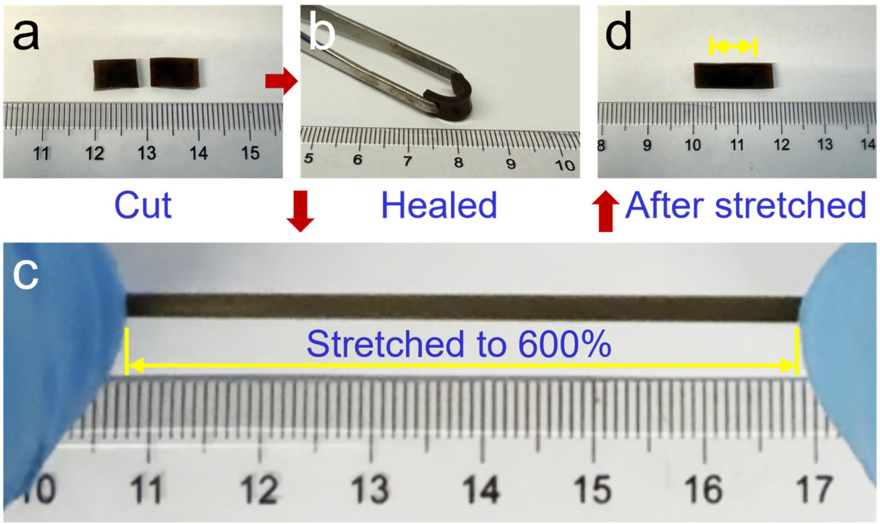

Fig. S20 Optical images showing the detailed self-healing process of USEP-$M_{0.5}$: (a) cut, (b) healed, (c) stretched to 600%, and (d) after stretched.

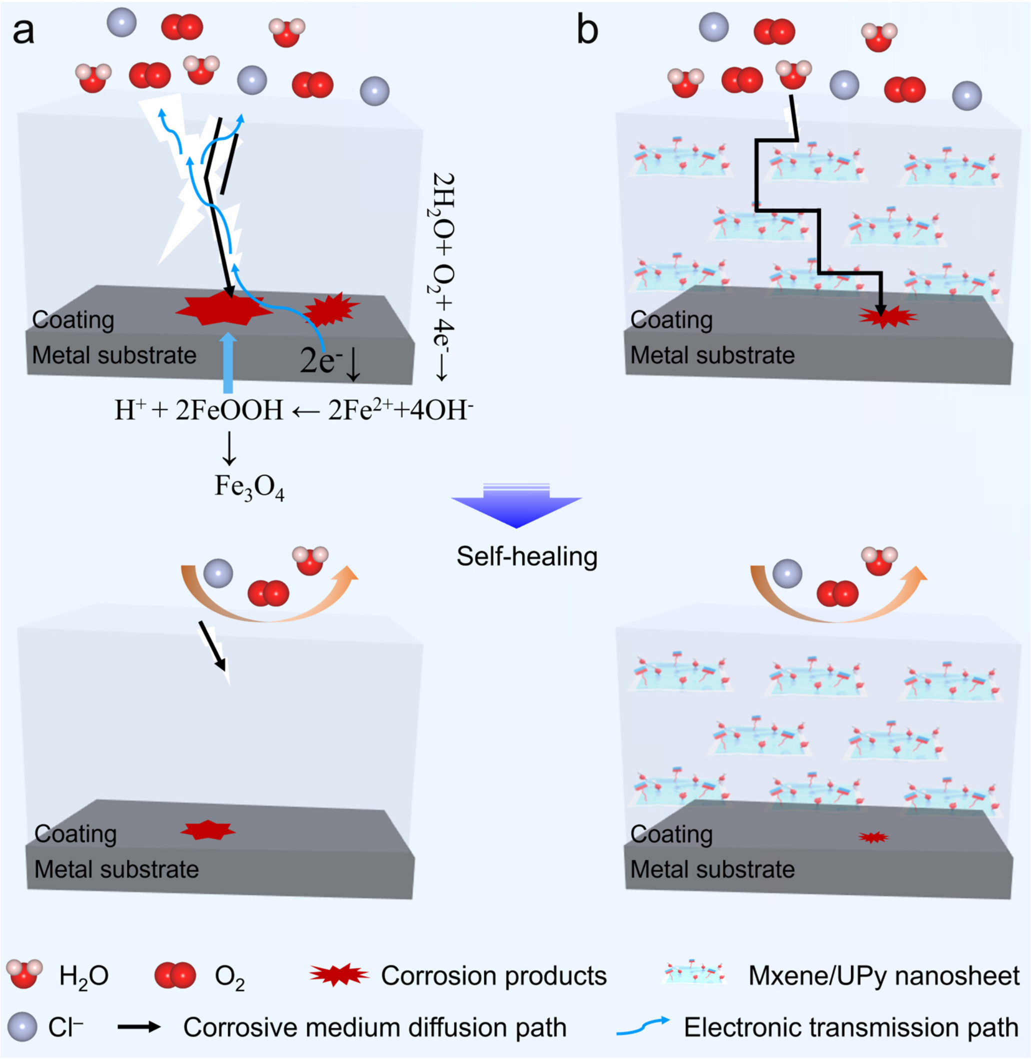

Fig. S21 Schematic diagram of coatings anticorrosion mechanism: (a) USEP and (b) USEP-$M_{0.5}$.

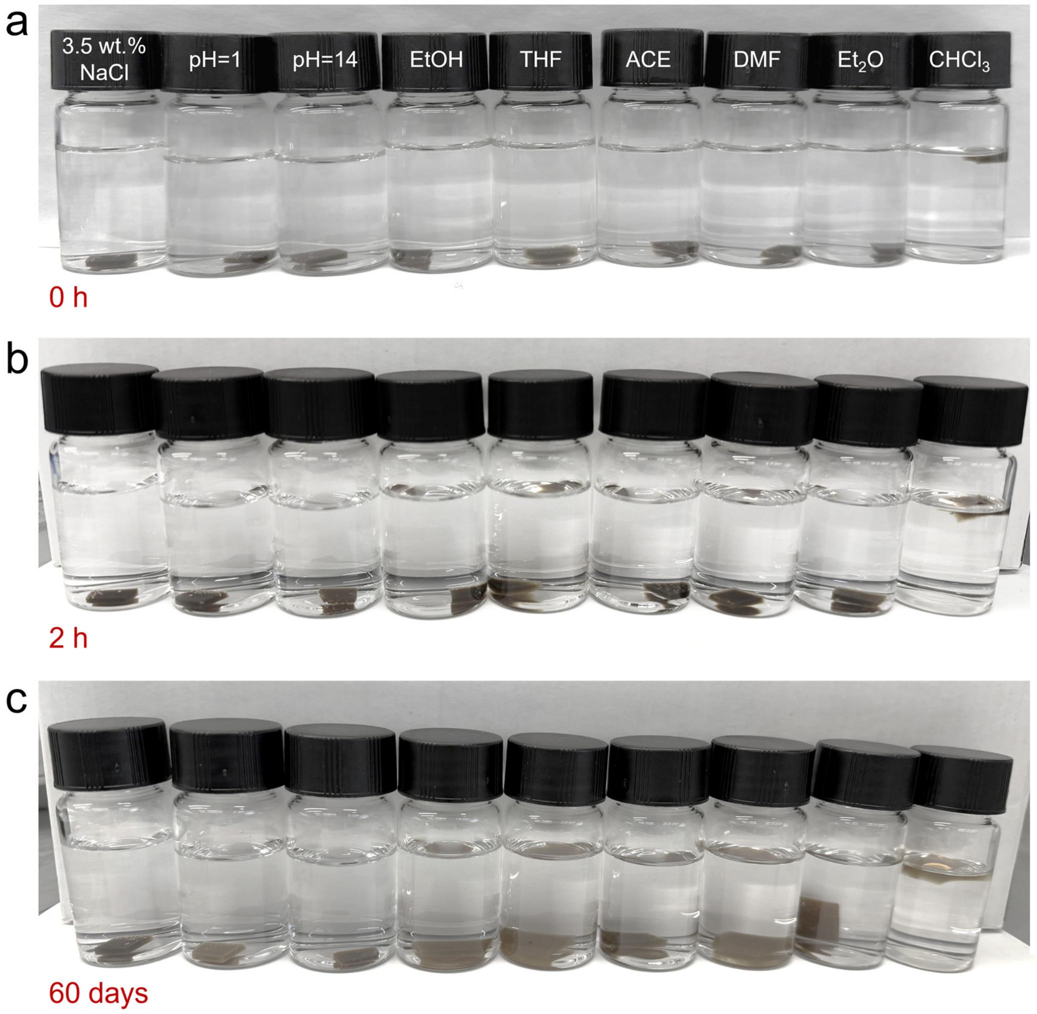

Fig. S22 Optical images of USEP-$M_{0.5}$ after immersion in various solutions at room temperature for different times: (a) 0 h, (b) 2 h, and (c) 60 days.

To assess the exceptional chemical stability of the epoxy adaptive network, USEP-$M_{0.5}$ was immersed in various substances, including salt water (3.5 wt.% NaCl), hydrochloric acid (HCl, pH = 1), sodium hydroxide (NaOH, pH = 14), and organic solvents [ethanol (EtOH), tetrahydrofuran (THF), acetone (ACE), N,N-dimethylformamide (DMF), ethyl ether (Et_2O), and trichloromethane ($CHCl_3$)]. After 2 h of immersion, the epoxy showed no changes when exposed to salt water, strong acids, or strong bases. The epoxy immersed in the organic solvent exhibited slight swelling, with

a minor increase in mass (0.5%-2.1%) compared to the original, as shown in Fig. S23. The mass after immersion was measured by wiping the solvent from the epoxy surface with filter paper and weighing it. The epoxy can return to its original mass after being dried at 60°C. The epoxy samples showed no change in volume after 60 days of immersion in salt water, strong acid, or strong alkali. During this period, the epoxy immersed in the organic solvents remained intact, demonstrating excellent water resistance and chemical stability.

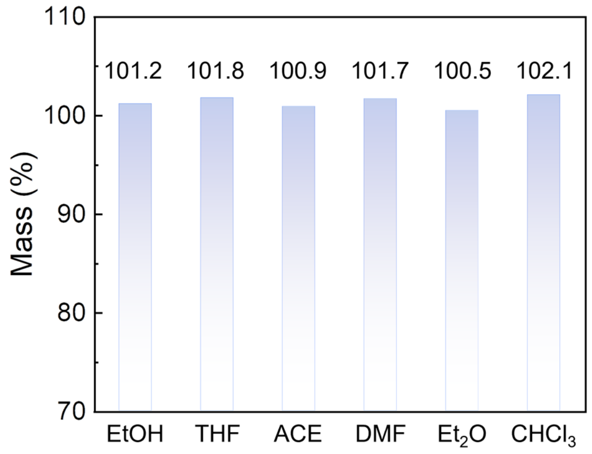

Fig. S23 Mass of the USEP-$M_{0.5}$ after immersion in different organic solvents at room temperature for 2 h. The epoxy can recover to their original mass after drying at 60°C.

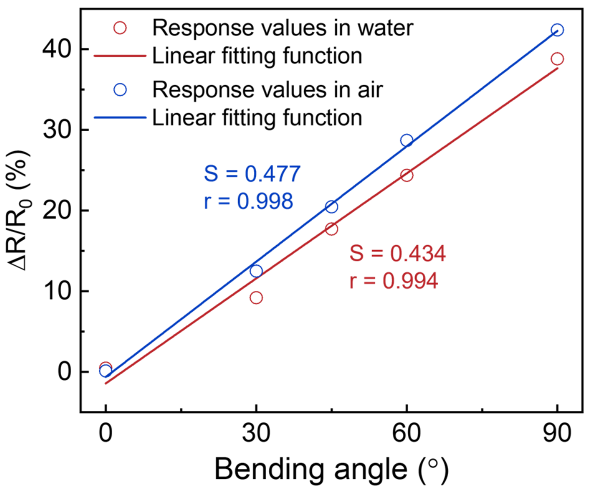

Fig. S24 Sensitivity of the M/EP/M sensor under different bending angles in air and water.

Supplementary Tables

Table S1 Results of the multiplication of the signs of each cross-peak in 2DCOS synchronous and asynchronous spectra of USEP-$M_{0.5}$.

1701.5 cm^{-1}	+	–	–	
1716.6 cm^{-1}	+	+		
3325.9 cm^{-1}	+			
3460.4 cm^{-1}				
**Wavenumber**	1701.5 cm^{-1}	1716.6 cm^{-1}	3325.9 cm^{-1}	3460.4 cm^{-1}

Table S2 Comparison of ultimate tensile strength, elongation at break, toughness, and Young's modulus of epoxy composites.

**Samples**	**Ultimate tensile strength (MPa)**	**Elongation at break (%)**	**Toughness (MJ m^{-3})**	**Young's modulus (MPa)**
USEP	20.19 ± 2.41	812.71 ± 11.28	94.41 ± 7.63	90.52 ± 6.83
USEP-$M_{0.2}$	28.41 ± 1.44	830.90 ± 13.42	134.78 ± 12.64	125.61 ± 9.42
USEP-$M_{0.5}$	34.50 ± 2.34	864.72 ± 15.81	210.75 ± 16.28	160.32 ± 13.52
USEP-$M_{1.0}$	31.41 ± 1.86	795.29 ± 14.72	163.85 ± 14.41	159.14 ± 12.38

Table S3 Mechanical properties of toughened epoxies reported in the literature.

Toughening mechanism	Ultimate tensile strength (MPa)	Elongation at break (%)	Toughness (MJ m^{-3})	Reference
Nanoscale phase separation	41.0	375.0	108.4	Main text ref. 2
Hyperbranched topological structure	104.5	5.65	3.58	Main text ref. 6
B-N coordination boronic esters	45.5	321.7	119.2	Main text ref. 7
H-bonds	25.6	112.4	19.5	Main text ref. 11
	14.7	184.0	7.5	
Network topology	48.0	9.7	4.7	Main text ref. 15
Sacrificial reversible iron-catechol cross-links	21.9	172	22.0	Main text ref. 22
Rubber filler	39.0	11.0	5.9	Main text ref. 36
Hyperbranched structure	114.43	3.57	2.15	Main text ref. 37
Solvent evaporation	38.9	80.0	29.0	Main text ref. 38
Block copolymer	63.7	8.0	3.5	Main text ref. 39
Rubber filler	76.7	11.3	4.3	Main text ref. 40
Inorganic filler	72.0	4.8	1.8	Main text ref. 41
Inorganic filler	97.1	4.4	2.5	Main text ref. 42
Network topology	51.3	17.9	6.7	Main text ref. 43
Network topology	38.9	42.8	14.6	Main text ref. 44
Thermoplastics	81.0	2.6	1.7	Main text ref. 45
Network topology	40.0	60.0	22.0	Main text ref. 46
Hierarchical crosslink structure	56.3	280.0	142.0	Main text ref. 47
Multi-dynamic bonds and nanosheets	20.19	812.71	94.41	This work
	28.4	830.9	134.78	
	34.5	864.7	210.75	
	31.41	795.2	163.85	

Table S4 Comparison of ultimate tensile strength, elongation at break, self-healing efficiency, and self-healing conditions of epoxy composites under different self-healing times.

Samples	Ultimate tensile strength (MPa)	Elongation at break (%)	Self-healing efficiency (%)	Self-healing conditions
USEP	18.65 ± 1.68	750.46 ± 10.68	92.37 ± 2.14	RT., 2 h
USEP-$M_{0.2}$	25.94 ± 2.23	754.31 ± 12.34	91.31 ± 1.88	RT., 2 h
USEP-$M_{0.5}$	31.17 ± 2.64	772.70 ± 11.39	90.35 ± 1.76	RT., 2 h
USEP-$M_{1.0}$	26.52 ± 2.71	672.47 ± 12.22	84.43 ± 1.89	RT., 2 h
USEP	16.98 ± 1.35	580.21 ± 19.67	84.10 ± 2.59	0℃, 2 h
USEP-$M_{0.2}$	23.31 ± 1.82	581.54 ± 17.38	82.05 ± 2.66	0℃, 2 h
USEP-$M_{0.5}$	28.12 ± 2.14	596.30 ± 21.34	81.51 ± 3.01	0℃, 2 h
USEP-$M_{1.0}$	21.26 ± 2.09	469.31 ± 25.61	67.69 ± 5.48	0℃, 2 h
USEP-$M_{0.5}$	9.94 ± 0.94	228.35 ± 7.67	28.81 ± 0.74	RT., 30 min
USEP-$M_{0.5}$	21.66 ± 1.94	518.93 ± 11.57	62.78 ± 1.18	RT., 60 min
USEP-$M_{0.5}$	27.96 ± 2.28	682.80 ± 13.34	81.04 ± 1.42	RT., 90 min

[*] RT. denotes room temperature (25℃).

Table S5 Comparison of ultimate tensile strength, elongation at break, toughness, and self-healing efficiency of USEP-$M_{0.5}$ repaired at 25°C for 2 h with different "damage/healing" cycles.

Samples	Ultimate tensile strength (MPa)	Elongation at break (%)	Toughness (MJ m^{-3})	self-healing efficiency (%)
USEP-$M_{0.5}$	34.50	864.72	210.7	/
1st	31.17	772.70	150.98	90.35
2st	31.21	773.23	162.58	90.46
3st	32.04	778.56	165.13	92.87
4st	30.84	767.31	145.32	89.39
5st	31.12	771.40	157.72	90.20

Table S6 Comparison of the mechanical properties and self-healing capabilities of the proposed USEP-$M_{0.5}$ with those of self-healing epoxies reported in the literature.

Healing motif	Ultimate tensile strength (MPa)	Elongation at break (%)	Toughness (MJ m^{-3})	Self-healing efficiency (%)	Self-healing conditions	Reference
Dynamic bonds	3.8	27.5	/	90.0	110°C, 45 min, 5 MPa	Main text ref. 8
	10.1	2.9	/	42.0		
H-bonds	10.6	173.0	6.3	72.1	RT., 5 min	Main text ref. 11
Ionic liquid/SAP	2.2	25.0	/	/	RT., 14 h	Main text ref. 48
Disulfide bonds	0.21	100.0	/	91.0	RT., 24 h	Main text ref. 49
Diels-Alder	44.0	65.0	/	105.8	60°C	Main text ref. 50
Dynamic imine bond	50.2	5.8	2.0	80.9	80°C, 3 min	Main text ref. 51
				100.0	80°C, 8 min	
Dynamic bonds	43.2	25.0	5.7	90.0	RT., 24 h	Main text ref. 52
H-bonds + disulfide bonds	34.5	864.72	210.75	90.35	RT., 2 h	This work

* RT. denotes room temperature (25°C).

Table S7 Oxygen transmission rates and water vapor transmission rates of epoxy composites.

Samples	Oxygen transmission rates (cm^3 m^{-2} d^{-1})	Water vapor transmission rates (g m^{-2} d^{-1})
USEP	9664.1 ± 2890.2	105874.3 ± 35765.6
USEP-$M_{0.2}$	78.2 ± 29.6	919.5 ± 247.3
USEP-$M_{0.5}$	21.7 ± 6.6	554.2 ± 197.5
USEP-$M_{1.0}$	24.4 ± 8.3	611.3 ± 214.4

Table S8 Shear strength of epoxy composites as an adhesive on different metal substrates.

Samples	Shear strength (MPa)	
	Q235 carbon steel	Aluminum
USEP	7.11 ± 0.62	6.23 ± 0.58
USEP-$M_{0.2}$	8.76 ± 0.79	7.62 ± 0.64
USEP-$M_{0.5}$	9.58 ± 0.82	8.44 ± 0.77
USEP-$M_{1.0}$	9.05 ± 0.76	7.98 ± 0.62

Supplementary References

[1] Y. Song, Y. Liu, T. Qi, G. L. Li, *Angew. Chem. Int. Edit.* **2018**, *57*, 13838-13842.

[2] Y. Wang, T. Li, P. Ma, S. Zhang, H. Zhang, M. Du, Y. Xie, M. Chen, W. Dong, W. Ming, *ACS Nano* **2018**, *12*, 6228-6235.

[3] G. Kresse, D. Joubert, *Phys. Rev. B* **1999**, *59*, 1758-1775.

[4] P. E. Blöchl, *Phys. Rev. B* **1994**, *50*, 17953-17979.

[5] J. P. Perdew, J. A. Chevary, S. H. Vosko, K. A. Jackson, M. R. Pederson, D. J. Singh, C. Fiolhais, *Phys. Rev. B* **1992**, *46*, 6671-6687.

[6] J. P. Perdew, K. Burke, M. Ernzerhof, *Phys. Rev. Lett.* **1996**, *77*, 3865-3868.

[7] S. Grimme, J. Antony, S. Ehrlich, H. Krieg, *J. Chem. Phys.* **2010**, *132*, 154104.
